# Predisposing and Precipitating Factors Associated With Delirium: A Systematic Review

**DOI:** 10.1001/jamanetworkopen.2022.49950

**Published:** 2023-01-06

**Authors:** Cora H. Ormseth, Sara C. LaHue, Mark A. Oldham, S. Andrew Josephson, Evans Whitaker, Vanja C. Douglas

**Affiliations:** 1 Department of Emergency Medicine, University of California, San Francisco; 2 Department of Neurology, University of California, San Francisco; 3 Department of Psychiatry, University of Rochester Medical Center, Rochester, New York; 4 University of California, San Francisco, School of Medicine

## Abstract

**Importance:**

Despite discrete etiologies leading to delirium, it is treated as a common end point in hospital and in clinical trials, and delirium research may be hampered by the attempt to treat all instances of delirium similarly, leaving delirium management as an unmet need. An individualized approach based on unique patterns of delirium pathophysiology, as reflected in predisposing factors and precipitants, may be necessary, but there exists no accepted method of grouping delirium into distinct etiologic subgroups.

**Objective:**

To conduct a systematic review to identify potential predisposing and precipitating factors associated with delirium in adult patients agnostic to setting.

**Evidence Review:**

A literature search was performed of PubMed, Embase, Web of Science, and PsycINFO from database inception to December 2021 using search Medical Subject Headings (MeSH) terms *consciousness disorders*, *confusion*, *causality*, and *disease susceptibility*, with constraints of cohort or case-control studies. Two reviewers selected studies that met the following criteria for inclusion: published in English, prospective cohort or case-control study, at least 50 participants, delirium assessment in person by a physician or trained research personnel using a reference standard, and results including a multivariable model to identify independent factors associated with delirium.

**Findings:**

A total of 315 studies were included with a mean (SD) Newcastle-Ottawa Scale score of 8.3 (0.8) out of 9. Across 101 144 patients (50 006 [50.0%] male and 49 766 [49.1%] female patients) represented (24 015 with delirium), studies reported 33 predisposing and 112 precipitating factors associated with delirium. There was a diversity of factors associated with delirium, with substantial physiological heterogeneity.

**Conclusions and Relevance:**

In this systematic review, a comprehensive list of potential predisposing and precipitating factors associated with delirium was found across all clinical settings. These findings may be used to inform more precise study of delirium’s heterogeneous pathophysiology and treatment.

## Key Points

**Question:**

What predisposing and precipitating factors are associated with delirium?

**Findings:**

In this systematic review of 315 studies of delirium representing 101 144 patients across all settings and populations, 33 predisposing and 112 precipitating factors were associated with delirium. Putative pathophysiological mechanisms associated with these factors were heterogeneous.

**Meaning:**

This study found physiological heterogeneity represented across studies, suggesting that delirium may not be not restricted to a singular physiological account.

## Teaser

This systematic review investigates predisposing and precipitating factors associated with delirium.

## Introduction

Delirium is an acute and often fluctuating disturbance in attention and awareness that is extremely common among hospitalized older adults, with an incidence of 29% to 64% in general medical wards, 50% after high-risk surgical procedures, and up to 75% in patients receiving mechanical ventilation in the intensive care unit.^1,2,3^ Delirium is associated with adverse outcomes, including increased risk of falls, functional decline, dementia, prolonged hospitalization, institutionalization, and death, at an annual cost of $38 billion to $152 billion in the US.^4,5^

Heterogeneous delirium phenotypes and the many predisposing factors, precipitants, and pathophysiological mechanisms associated with delirium make this condition challenging to identify, manage, and study.^6^ Despite its heterogeneous etiologies, delirium remains a blanket diagnosis, so it is unsurprising that treatment trials to address delirium of all causes have largely been ineffective.^7^ The understanding of delirium etiology is a major unmet need.^8,9^ Currently, there is no consistently used comprehensive framework for categorizing delirium etiologies, although multiple models have been proposed.^6,7,10,11,12,13^

While the heterogeneity of the literature on risk factors associated with delirium precludes a meta-analysis, this systematic review aimed to identify potential predisposing and precipitating factors associated with delirium across all clinical settings and patient populations to provide the basis for a framework for standardizing delirium classification into major pathophysiological categories. We believe this foundation may be necessary to develop a precision medicine approach to delirium in which etiology and pathophysiology inform research and therapeutic strategies.^14^

## Methods

This systematic review was registered on the International Prospective Register of Systematic Reviews database (PROSPERO) on April 28, 2020 (CRD42020147254). We followed the Preferred Reporting Items for Systematic Reviews and Meta-analyses (PRISMA) reporting guideline.

### Literature Search

Literature search strategies were developed in collaboration with a medical librarian with expertise in systematic review searching (E.W.). Medical Subject Headings (MeSH) and text words related to risk factors and precipitants associated with delirium were used to form search terms. Confusion was chosen as a search term because it includes the term delirium in the MeSH category hierarchy; given the variable terminology used for delirium, the more inclusive term was chosen. The transcript of the PubMed strategy follows: ((((“consciousness disorders”[Mesh] OR confusion[MeSH Terms]) AND (causality[MeSH Terms] OR disease susceptibility[MeSH Terms]) AND (“Cohort Studies”[Mesh] OR cohort OR “Case-Control Studies”[Mesh] OR case-control OR “Longitudinal Studies”[Mesh] OR longitudinal OR “Prospective Studies”[Mesh] OR prospective OR “logistic models”[mh] OR “logistic regression”))).

This search strategy was adapted for other databases. We searched PubMed, Embase, Web of Science, and PsycINFO without date or language limitations. Additionally, reference lists of included studies and relevant reviews identified by our search were manually searched for relevant articles. Databases were searched from inception to December 2021. Literature search results were uploaded to Covidence, a web-based software management program for coordinating systematic literature reviews (Veritas Health Innovation).

### Study Selection

In July 2019, two authors (C.H.O. and S.C.L.) tested inclusion and exclusion criteria on a sample of 195 articles through the online software Rayyan (Qatar Computing Research Institute). Interrater reliability was 0.61 by Cohen κ. Discrepancies were reviewed by discussion. Subsequently, 1 author (C.H.O.) screened titles and abstracts for eligibility criteria. Studies were advanced to full text review if they met the following criteria: published in English, prospective cohort or case-control study design, at least 50 participants, study population consisting of adults only, and primary or secondary objective to identify risk factors associated with delirium or delirium assessed as a primary or secondary outcome. From November 2019 to October 2022, two authors (C.H.O. and S.C.L.) independently screened the full texts for articles meeting eligibility criteria. Interrater reliability was 0.64 by Cohen κ. Studies were included in the review if they met the following criteria: delirium assessment in person by a physician or trained research personnel using a reference standard and results including a multivariable model to identify independent factors associated with delirium. Reference standards were the *Diagnostic and Statistical Manual of Mental Disorders* (Third Edition) (*DSM-III*), *Diagnostic and Statistical Manual of Mental Disorders* (Fourth Edition) (*DSM-IV*), *Diagnostic and Statistical Manual of Mental Disorders* (Fifth Edition) (*DSM-5*), Confusion Assessment Method (CAM), CAM for the Intensive Care Unit (CAM-ICU), Delirium Rating Scale (DRS), and DRS-Revised-98. Studies were excluded if delirium was diagnosed by bedside nurse, the article did not specify who diagnosed delirium, other delirium diagnostic tools not listed above were used, or the study was restricted to patients with COVID-19. Studies reporting data from the same cohort were excluded unless they described distinct predisposing or precipitating factors; in such cases, the most recent publication was included and total participants were counted once. We used this method to ensure that all distinct predisposing or precipitating factors identified in the studies were represented without falsely increasing the total count of studies or participants in our tables. The senior author (V.C.D.) adjudicated discrepancies.

### Outcome Measures

Our primary outcomes were predisposing and precipitating factors associated with delirium. Predisposing factors were defined as any patient characteristic that was more prevalent among patients with delirium, with a *P* value < .05 on multivariable analysis, and that preceded delirium onset by at least 1 month, including preoperative findings associated with postoperative delirium. When duration was not specified, factors were reviewed individually and assigned the most likely temporal association with delirium (eg, prescription medications at study enrollment were considered a predisposing factor). Precipitating factors were defined as any event occurring in the month prior to onset of delirium, usually of an acute or subacute nature, and more prevalent among patients with delirium, with a *P* value < .05 on multivariable analysis. Because predisposing and precipitating factors were identified using strict criteria, data extraction was performed independently by 2 authors (C.H.O. and S.C.L.) without calculating interrater reliability. The senior author (V.C.D.) adjudicated discrepancies. Consistent with the recent multisociety position statement,^15^ we chose 1 month as a time point given that delirium is the primary expression of a rapidly developing (<4 weeks) acute encephalopathy; factors present more than 1 month prior to delirium onset were unlikely to be associated with the outcome. It is possible that some factors were both predisposing and precipitating (anemia and pain, for example) depending on timing (chronic vs acute). In such instances, they were listed as both precipitating and predisposing factors. Rarely, a study identified a variable that appeared to be associated with protection against the development of delirium. These findings are also presented. It is possible that certain factors were positively associated with delirium in some studies but not in others; because of heterogeneity, meta-analysis was not possible, and therefore such negative associations were not captured.

### Quality Assessment

Risk of bias was assessed by 2 reviewers (C.H.O. and S.C.L.) using the Newcastle-Ottawa Scale (NOS).^16^ The NOS is a validated tool for quality assessment that contains 8 items categorized into 3 domains (selection, comparability, and outcome or exposure). Each favorable attribute earns 1 to 2 points, for a minimum of 0 points and a maximum of 9 points. Owing to stringent inclusion and exclusion criteria, all studies in this review scored between 7 and 9, representing a low risk of bias.

### Data Extraction

We developed a standardized data-extraction protocol using Covidence software. The following variables were extracted by 2 reviewers (C.H.O. and S.C.L.): citation, country, year, study design, period of data collection, study setting (hospital, nursing home, or community dwelling), number of study sites, study environment (hospital ward, emergency department, or intensive care unit), patient type (general medical, postoperative, or cardiac), participant inclusion and exclusion criteria, personnel measuring delirium, delirium assessment tool, assessment frequency, number of participants, number of participants with delirium, participant sex, participant age, delirium incidence and prevalence, predisposing factors, precipitating factors, and whether biomarkers were evaluated. Race data were not extracted or evaluated in this study because race and ethnicity were not consistently reported across studies. However, race data are presented from 1 study (Pisani et al^17^) and findings on race summarized from 2 studies (Hsieh et al^18^and Khan et al^19^) to illustrate how a factor identified in a single study may have been eliminated in a meta-analysis. Pisani et al^17^ did not specify which racial or ethnic groups were included or how race or ethnicity were reported. In Hsieh et al,^18^ race and ethnicity were reported by patients, and race and ethnicity categories were Black, White, multiracial, and other and Hispanic ethnicity; in Khan et al,^19^ race and ethnicity were reported by the patient or caregiver, and race and ethnicity categories were African American and Caucasian.

### Data Analysis

Predisposing and precipitating factors were grouped when definitions overlapped. For example, cognitive impairment and dementia were defined heterogeneously across studies, some using a preexisting diagnosis and others using scores on cognitive tests, such as the Mini-Mental State Examination, Telephone Interview for Cognitive Status, Mini-Cog, or Montreal Cognitive Assessment. These predisposing factors were grouped as cognitive impairment or dementia. Predisposing and precipitating factors were presented in order by the number of participants included in studies in which each factor was examined. Precipitating factors were further grouped by major medical category: surgical factor, systemic illness and organ dysfunction, metabolic abnormality, pharmacology, iatrogenic and environmental factor, trauma, and biomarker and neurotransmitter. For each precipitating factor, we identified putative mechanisms underlying delirium pathophysiology based on prior studies and reviews on theoretical mechanisms.^6,13^ Factors were then grouped based on their most relevant underlying mechanisms.

## Results

### Identification of Studies

The search yielded 4597 articles, of which 471 were duplicates (eFigure in Supplement 1). Of 4126 articles screened by title and abstract, 2729 were excluded. The 1397 remaining articles were advanced to full text screen. Of these, 1082 articles were excluded. Reasons for exclusion were no diagnosis by physician or research personnel (428 studies), no characterization of predisposing or precipitating factors (338 studies), retrospective study design (86 studies), not diagnosed by reference standard (108 studies), no multivariable model (43 studies), delirium assessment not conducted in person (25 studies), not cohort or case-control design (20 studies), sample included less than 50 patients (12 studies), abstract only (18 studies), cohort previously reported (3 studies), and pediatric population (1 studies). Ultimately, 315 studies^17,18,19,20,21,22,23,24,25,26,27,28,29,30,31,32,33,34,35,36,37,38,39,40,41,42,43,44,45,46,47,48,49,50,51,52,53,54,55,56,57,58,59,60,61,62,63,64,65,66,67,68,69,70,71,72,73,74,75,76,77,78,79,80,81,82,83,84,85,86,87,88,89,90,91,92,93,94,95,96,97,98,99,100,101,102,103,104,105,106,107,108,109,110,111,112,113,114,115,116,117,118,119,120,121,122,123,124,125,126,127,128,129,130,131,132,133,134,135,136,137,138,139,140,141,142,143,144,145,146,147,148,149,150,151,152,153,154,155,156,157,158,159,160,161,162,163,164,165,166,167,168,169,170,171,172,173,174,175,176,177,178,179,180,181,182,183,184,185,186,187,188,189,190,191,192,193,194,195,196,197,198,199,200,201,202,203,204,205,206,207,208,209,210,211,212,213,214,215,216,217,218,219,220,221,222,223,224,225,226,227,228,229,230,231,232,233,234,235,236,237,238,239,240,241,242,243,244,245,246,247,248,249,250,251,252,253,254,255,256,257,258,259,260,261,262,263,264,265,266,267,268,269,270,271,272,273,274,275,276,277,278,279,280,281,282,283,284,285,286,287,288,289,290,291,292,293,294,295,296,297,298,299,300,301,302,303,304,305,306,307,308,309,310,311,312,313,314,315,316,317,318,319,320,321,322,323,324,325,326,327,328,329,330,331^ were included in this review.

### Study Characteristics

Of 315 included studies,^17,18,19,20,21,22,23,24,25,26,27,28,29,30,31,32,33,34,35,36,37,38,39,40,41,42,43,44,45,46,47,48,49,50,51,52,53,54,55,56,57,58,59,60,61,62,63,64,65,66,67,68,69,70,71,72,73,74,75,76,77,78,79,80,81,82,83,84,85,86,87,88,89,90,91,92,93,94,95,96,97,98,99,100,101,102,103,104,105,106,107,108,109,110,111,112,113,114,115,116,117,118,119,120,121,122,123,124,125,126,127,128,129,130,131,132,133,134,135,136,137,138,139,140,141,142,143,144,145,146,147,148,149,150,151,152,153,154,155,156,157,158,159,160,161,162,163,164,165,166,167,168,169,170,171,172,173,174,175,176,177,178,179,180,181,182,183,184,185,186,187,188,189,190,191,192,193,194,195,196,197,198,199,200,201,202,203,204,205,206,207,208,209,210,211,212,213,214,215,216,217,218,219,220,221,222,223,224,225,226,227,228,229,230,231,232,233,234,235,236,237,238,239,240,241,242,243,244,245,246,247,248,249,250,251,252,253,254,255,256,257,258,259,260,261,262,263,264,265,266,267,268,269,270,271,272,273,274,275,276,277,278,279,280,281,282,283,284,285,286,287,288,289,290,291,292,293,294,295,296,297,298,299,300,301,302,303,304,305,306,307,308,309,310,311,312,313,314,315,316,317,318,319,320,321,322,323,324,325,326,327,328,329,330,331^ including 101 144 patients (50 006 [50.0%] male and 49 766 [49.1%] female patients; 24 015 patients with delirium), date of publication spanned 29 years, from 1992 to 2021 (Table 1). Studies were conducted in 40 different countries, with the plurality in the United States (86 studies^17,18,19,20,21,22,28,29,32,37,44,46,54,55,61,66,81,82,84,102,103,106,113,115,116,118,120,125,126,129,136,138,140,142,143,147,148,149,150,161,162,163,173,175,176,177,178,179,187,188,191,192,193,199,201,202,208,210,215,216,220,222,223,224,228,235,238,242,246,247,248,251,252,254,257,261,262,273,275,280,282,295,296,297,308,317^ [27.3%]), followed by China (48 studies^23,40,78,85,86,87,88,114,117,130,132,133,137,141,166,181,182,184,185,189,190,204,205,206,217,230,236,237,243,255,276,281,303,304,305,306,307,316,318,319,320,322,324,325,326,327,328,329^ [15.2%]) and the Netherlands (24 studies^48,63,67,70,100,110,111,128,154,167,264,265,287,288,290,291,292,293,294,301,311,313,314,323^ [7.6%]). Most studies were prospective cohort studies (296 studies^17,18,19,20,21,22,23,24,25,26,27,28,29,30,31,32,33,34,35,36,37,38,39,40,41,42,43,44,45,46,47,49,50,51,53,54,55,56,58,59,61,63,64,65,66,67,68,69,70,71,72,73,74,75,76,78,79,80,81,82,83,84,85,86,87,88,89,91,92,94,95,96,97,98,99,100,101,103,104,105,106,107,108,109,110,111,112,113,114,115,116,117,118,119,120,121,123,124,125,126,127,128,129,130,131,132,133,134,135,136,137,138,139,140,141,142,143,144,145,146,147,148,149,150,151,152,153,154,155,156,157,158,159,160,161,162,163,164,165,167,168,169,170,171,173,174,175,176,177,178,179,180,181,182,183,184,185,186,187,188,189,190,191,193,194,195,196,197,198,199,200,201,202,203,204,205,206,208,209,210,211,212,213,214,215,217,218,219,220,221,222,223,224,225,226,227,228,229,230,231,232,233,234,235,236,237,238,239,240,241,242,243,244,245,246,247,248,249,250,251,252,253,254,255,256,257,258,259,260,261,262,263,264,265,266,267,269,270,271,272,273,274,275,276,277,278,279,280,281,283,284,285,286,287,288,289,290,291,292,293,294,295,296,297,298,299,301,302,303,304,305,306,307,308,309,310,311,312,313,314,315,316,317,318,319,320,321,323,324,325,326,327,328,329,330,331^ [94.0%]) conducted in a hospital setting (310 studies^17,18,19,20,21,22,23,24,25,26,27,28,30,31,32,33,34,35,36,37,38,39,40,41,42,43,44,45,46,47,48,49,50,52,53,54,55,56,57,58,59,60,61,62,63,64,65,66,67,68,69,70,71,72,73,74,75,76,77,78,79,80,81,82,83,84,85,86,87,88,89,90,91,93,94,95,96,97,98,99,100,101,102,103,104,105,106,107,108,109,110,111,112,113,114,115,116,117,118,119,120,121,122,123,124,125,126,127,128,129,130,131,132,133,134,135,136,137,138,139,140,141,142,143,144,145,146,147,148,149,150,151,152,153,154,155,156,157,158,159,160,161,162,163,164,165,166,167,168,169,170,171,172,173,174,175,176,177,178,179,180,181,182,183,184,185,186,187,188,189,190,191,192,193,194,195,196,197,198,199,200,201,202,203,204,205,206,207,208,209,210,211,212,213,214,215,216,217,218,219,220,221,222,223,224,225,226,227,228,229,230,232,233,234,235,236,237,238,239,240,241,242,243,244,245,246,247,248,249,250,251,252,253,254,255,256,257,258,259,260,261,262,263,264,265,266,267,268,269,270,271,272,273,274,275,276,277,278,279,280,281,282,283,284,285,286,287,288,289,290,291,292,293,294,295,296,297,298,300,301,302,303,304,305,306,307,308,309,310,311,312,313,314,315,316,317,318,319,320,321,322,323,324,325,326,327,328,329,330,331^ [98.4%]) with 1 study center (mean [SD] 1.6 [2.4] study centers). There were 143 studies^20,22,28,30,31,32,34,35,36,37,39,41,46,48,51,55,56,59,62,63,66,67,68,70,71,73,75,80,81,82,84,86,87,90,92,95,97,100,102,106,108,109,110,111,112,117,120,123,124,127,131,132,135,137,138,140,144,147,148,149,150,151,152,154,155,161,167,168,170,173,174,176,177,178,179,182,185,186,187,191,194,198,199,201,203,206,207,215,216,218,219,220,230,231,232,235,236,238,242,244,251,255,256,257,258,261,263,264,265,266,270,273,276,277,278,280,281,282,285,289,290,291,292,293,294,295,296,297,299,303,307,308,312,313,314,320,322,324,325,326,328,329,331^ (45.4%) restricted to older adult populations (patients aged ≥65 years). The mean (SD) NOS was 8.3 (0.8). A list of included studies with number of participants and NOS score is included in eTable in Supplement 1). There were 138 studies^17,18,19,20,21,24,25,26,28,31,36,38,43,46,47,48,51,53,54,58,61,63,65,66,67,68,70,71,73,78,81,82,86,87,92,97,98,100,101,102,103,105,110,111,112,114,118,119,124,126,127,129,132,136,137,140,141,142,143,147,150,151,152,153,154,157,158,159,160,161,162,163,167,169,175,176,177,178,181,182,187,188,189,190,191,192,193,194,200,201,202,207,210,215,216,222,223,224,228,235,238,242,244,246,250,252,257,261,263,264,269,271,272,273,274,275,279,280,284,285,290,291,292,293,294,295,296,297,299,301,305,308,317,322,323,324,325,326^ (43.8%) that excluded participants due to a language barrier (participants were excluded if they did not speak the same language that delirium assessors used).

**Table 1.** Characteristics of Included Studies

Characteristic	Studies, No. (%) (N = 315)
Publication date, range, y	1992-2021
Data collection date, range, y	
Start	1987-2020
End	1989-2020
Countries in which study was conducted, No.	40
Study design	
Cohort	296 (94.0)
Case control	19 (6.0)
Study setting	
Hospital	310 (98.4)
Nursing home	2 (0.6)
Community dwelling	3 (1.0)
Study centers, mean (SD), No.	1.6 (2.4)
Study environment	
Hospital ward	213 (67.6)
Intensive care unit	90 (28.6)
Emergency department	7 (2.2)
Other	5 (1.6)
Patient type	
General medical	98 (31.1)
Postoperative	151 (47.9)
Cardiac	40 (12.7)
Other	26 (8.3)
Older adult population (aged ≥65 y)	143 (45.4)
Total participants, range, No.	51-5781
Sex, mean (SD), %	
Male	50.0 (16.8)
Female	49.1 (16.6)
Quality assessment points, mean (SD)	8.3 (0.8)
Delirium measured by physician	139 (44.1)
Delirium assessment tool	
CAM	174 (55.2)
CAM-ICU	74 (23.5)
* DSM-III*	11 (3.5)
* DSM-IV*	39 (12.4)
* DSM-5*	13 (4.1)
DRS	2 (0.6)
DRS-Revised-98	2 (0.6)
At least daily assessments	222 (70.5)
Delirium, range, No.	
Incidence	3.1-88.7
Prevalence	3.4-70.3

Abbreviations: CAM, Confusion Assessment Method; CAM-ICU, Confusion Assessment Method for the Intensive Care Unit; DRS, Delirium Rating Scale; *DSM-III*, *Diagnostic and Statistical Manual of Mental Disorders* (Third Edition); *DSM-IV*, *Diagnostic and Statistical Manual of Mental Disorders* (Fourth Edition); *DSM-5*, *Diagnostic and Statistical Manual of Mental Disorders* (Fifth Edition).

### Delirium Assessment

Delirium was measured by research personnel in 171 studies^17,18,19,20,22,26,27,28,29,34,35,36,37,38,41,44,46,48,49,51,54,58,61,65,66,67,68,74,75,78,79,81,82,84,85,88,91,92,94,95,96,97,100,101,102,103,105,106,107,108,109,112,114,115,116,117,118,120,121,123,124,125,126,127,129,130,136,137,140,141,142,143,144,146,150,151,152,153,154,161,162,163,165,166,171,173,175,176,177,178,179,182,183,184,186,187,188,190,191,192,193,194,195,199,200,201,202,208,210,211,213,214,216,219,220,221,223,224,228,233,234,238,239,240,242,245,246,247,250,252,253,254,257,262,264,265,266,268,273,274,277,279,280,281,282,284,288,289,295,296,297,301,303,304,305,306,307,308,311,313,314,315,316,317,318,324,327,328,329,330,331^ (54.3%), a combination of research personnel and physicians in 5 studies^33,63,139,261,299^ (1.6%), and a physician in 139 studies.^21,23,24,25,30,31,32,39,40,42,43,45,47,50,52,53,55,56,57,59,60,62,64,69,70,71,72,73,76,77,80,83,86,87,89,90,93,98,99,104,110,111,113,119,122,128,131,132,133,134,135,138,145,147,148,149,155,156,157,158,159,160,164,167,168,169,170,172,174,180,181,185,189,196,197,198,203,204,205,206,207,209,212,215,217,218,222,225,226,227,229,230,231,232,235,236,237,241,243,244,248,249,251,255,256,258,259,260,263,267,269,270,271,272,275,276,278,283,285,286,287,290,291,292,293,294,298,300,302,309,310,312,319,320,321,322,323,325,326^ The most frequently used assessment tool was the CAM, which was used in 174 studies^20,21,22,23,25,27,28,29,30,31,34,35,36,37,39,44,46,51,55,56,57,58,59,61,62,63,66,68,70,73,74,75,79,80,81,82,83,89,90,91,92,95,96,97,98,100,101,102,103,104,106,112,118,119,120,122,123,124,127,129,131,132,133,134,137,138,139,140,144,146,147,148,149,150,151,152,153,154,161,164,166,167,172,173,174,175,176,177,178,183,185,187,188,190,192,193,194,195,197,198,199,200,203,205,206,207,212,213,214,216,217,218,220,221,222,225,226,227,232,237,238,239,240,241,242,243,244,245,247,248,253,257,258,261,263,264,265,272,273,275,276,277,280,281,282,289,290,291,292,293,294,295,296,297,298,299,300,303,308,309,310,313,314,317,319,320,321,322,325,326,328,329,330,331^ (55.2%). Delirium was assessed daily or more frequently in 222 studies^17,18,19,20,23,24,25,26,27,28,30,31,33,35,37,38,39,40,42,43,44,45,46,47,48,49,50,52,54,55,60,61,62,63,65,67,68,71,72,73,75,78,79,81,82,84,85,86,87,88,89,91,93,95,96,99,101,102,103,105,110,111,112,113,114,117,118,119,120,122,123,125,126,127,128,129,132,133,134,135,136,137,139,140,141,142,143,144,145,146,147,149,150,153,154,157,158,159,160,162,163,164,165,166,168,169,170,171,173,175,176,177,178,179,181,182,183,184,185,186,187,188,190,191,192,194,196,197,198,199,202,204,205,208,209,210,211,212,213,214,215,216,217,218,219,223,224,226,228,230,232,233,234,235,236,238,239,240,241,242,243,246,247,249,250,251,252,253,254,255,256,257,258,259,260,262,264,265,267,268,269,273,274,275,278,279,281,282,283,286,288,290,291,292,293,294,297,298,300,301,303,304,306,307,308,309,310,311,312,313,314,315,317,318,319,321,322,323,324,325,327,328^ (60.5%). Delirium incidence ranged from 42 of 1367 patients (3.1%)^45^ to 47 of 53 patients (88.7%),^113^ and prevalence ranged from 19 of 560 patients (3.4%)^39^ to 83 of 118 patients (70.3%).^201^

### Predisposing Factors Associated With Delirium

Predisposing factors associated with delirium are presented in Table 2, which lists the number of studies identifying each factor in multivariable analysis, number of participants included in those studies, and number of participants with delirium. A total of 33 predisposing factors were identified. Advanced age and cognitive impairment or dementia were identified in the most studies and participants; 112 studies^17,24,25,26,33,38,40,41,42,43,45,47,50,54,55,56,58,61,62,64,66,69,71,72,76,85,87,88,91,94,95,96,97,98,99,103,106,109,121,130,131,132,133,137,140,141,142,145,150,154,155,156,161,162,164,165,169,170,173,177,179,181,183,188,189,190,193,194,195,198,202,205,206,211,212,220,222,223,224,228,230,232,233,237,250,251,252,253,256,257,259,266,269,271,274,278,286,288,291,297,299,300,302,305,307,319,321,325,326,327,328,330^ with 50 418 participants (among whom 9147 patients had delirium) identified age and 130 studies^8,17,18,21,23,25,35,39,41,44,46,48,49,51,53,54,55,56,59,63,64,65,66,68,70,71,72,81,84,91,92,96,97,99,100,103,104,106,107,108,112,115,118,119,120,122,123,124,129,131,134,138,140,144,145,147,149,151,153,154,155,156,157,158,159,161,162,167,168,173,174,175,179,180,183,187,188,191,193,194,195,198,201,203,204,205,206,212,215,217,219,220,222,226,229,231,232,233,235,245,246,247,250,251,252,256,258,261,263,270,272,273,276,280,281,289,291,292,294,298,306,307,309,313,314,320,322,325,326,330^ with 42 124 participants (among whom 9617 patients had delirium) identified cognitive impairment or dementia as factors. The following predisposing factors were variably defined across studies: functional impairment, cardiovascular disease, central nervous system disorders, and psychiatric disorders. A wide range of cardiovascular disorders were associated with delirium risk, including heart failure, atrial fibrillation, coronary artery disease, hypertension, atherosclerosis, and peripheral arterial disease (including history of major amputation). White race was identified as a predisposing factor in 1 study^17^ with 309 participants; in this study, 51 participants (16.5%) were members of racial minority groups, 239 participants (77.3%) experienced delirium, and 10 variables were included in the multivariable model.^17^ Race was not identified as a predisposing factor associated with delirium in 2 larger studies.^18,19^ Hsieh et al^18^ included 191 Black participants (35.9%), 161 White participants (30.3%), 127 multiracial participants (23.9%), 53 participants of other racial categories (10.0%), and 187 participants of Hispanic ethnicity (35.2%), and Khan et al^19^ included 1767 Black participants (48.3%) and 1889 White participants (51.7%). A single study^18^ identified non-English language as a predisposing factor associated with delirium.

**Table 2.** The 33 Predisposing Factors Associated With Delirium

Predisposing factor	No.		
	Studies	Total participants	Participants with delirium
Advanced age	112	50 418	9147
Cognitive impairment^a^ or dementia	130	42 124	9617
Functional impairment (physical, vision, hearing, or frailty)	48	17 206	3679
Cardiovascular disease^b^	18	11 895	1422
Cumulative comorbidities^c^	26	10 528	2035
Central nervous system disorder^d^	24	9246	1861
Alcohol use	12	8100	1462
Male sex	15	4696	1112
Depression	19	4362	926
Lower educational attainment	8	3657	648
Malnutrition or undernutrition	9	2921	614
Diabetes	6	2775	1905
Tobacco use	7	2605	467
Anemia	5	2538	292
Psychiatric disorder or trait^e^	7	2138	326
Female sex	4	2134	636
Multiple medications	6	1287	323
Psychoactive medication	3	1074	177
Malignant neoplasm	2	846	188
Pain (chronic)	2	774	146
Pulmonary disease (OSA or COPD)	4	685	163
Poor sleep quality	4	655	154
Chronic kidney disease	1	560	63
Non-English language	1	532	241
Narcotic analgesic	1	500	57
White race	1	309	239
Low vitamin D	1	240	60
Anticholinergic	1	74	29
Biomarkers and genetics			
Biomarkers of neurodegeneration^f^	7	1114	237
SNVs in *DRD2* and *SLC6A3* gene	1	720	126
* APOE4*	2	169	76
AG haplotype of *GRIN3A* gene	1	102	41
*COMT* Val^127^ or Val^127^ genotype	1	89	17

Abbreviations: AG, adenine guanine; *APOE4*, apolipoprotein E4; COMT, catechol-O-methyltransferase; COPD, chronic obstructive pulmonary disease; *DRD2*, dopamine receptor 2 gene; *GRIN3A*, *N*-methyl-d-aspartate receptor 3A subunit gene; OSA, obstructive sleep apnea; *SLC6A3*, dopamine transporter gene on the 5p15.3 chromosome; SNV, single-nucleotide variation (formerly single-nucleotide polymorphism [SNP]); Val, valine.

^a^ Cognitive impairment was measured by Mini-mental State Examination, Telephone Interview for Cognitive Status, Mini-Cog, Montreal Cognitive Assessment, auditory verbal learning test, Trail Making Test, or Short Portable Mental Status Questionnaire.

^b^ Heart failure, mitral valve disease, cardiothoracic index, European System for Cardiac Operative Risk Evaluation score, American College of Cardiology and American Heart Association guidelines, atrial fibrillation, peripheral arterial disease, history of major amputation, coronary artery disease, atherosclerosis, and hypertension.

^c^ Measured by Cumulative Illness Rating Scale, American Society of Anesthesiologists class, Charlson Comorbidity Index, or National Surgical Quality Improvement Program Risk of Serious Complications.

^d^ Broadly defined, stroke, Parkinson disease, previous episode of delirium, and olfactory impairment.

^e^ Broadly defined, anxiety, neuroticism, and low conscientiousness.

^f^ Amyloid-β 1-42 levels in cerebrospinal fluid, diffusion tensor imaging abnormalities, white matter abnormalities, reduction in gray matter volume, and thinner cortex.

### Precipitating Factors Associated With Delirium

Precipitating factors associated with delirium are presented in Table 3. A total of 112 precipitating factors were identified. These were grouped into 8 major categories agnostic to pathophysiology: surgical factors, systemic illness or organ dysfunction, metabolic abnormalities, pharmacology, iatrogenic and environmental factors, trauma, biomarkers, and neurotransmitters. In 25 studies,^17,18,38,61,76,77,103,114,142,147,169,180,181,184,187,202,209,213,223,224,253,270,304,309,327^ scores for combined measures of organ dysfunction, such as the Acute Physiology and Chronic Health Evaluation II or Sequential Organ Failure Assessment, were associated with delirium. Many other studies identified individual components of such scores, such as hypoxemia, anemia, or leukocytosis, as factors associated with delirium. These precipitating factors and studies were therefore listed separately. Factors grouped based on their most relevant underlying mechanisms are displayed in Table 4.

**Table 3.** The 112 Precipitating Factors Associated With Delirium

Precipitating factor	No.		
	Studies	Total participants	Participants with delirium
Surgical factor			
Type of surgery^a^	23	15 864	2133
Intraoperative blood loss or transfusion	12	11 171	1250
Intraoperative hemodynamics	5	6684	442
Duration of operation	16	6172	1521
Postoperative complication, atrial fibrillation, or shock	6	3117	410
Prolonged time to operation	7	2457	816
Anesthesia type and depth^b^	4	772	179
No. of surgeries	3	610	125
Intraoperative fluids	2	295	138
Systemic illness or organ dysfunction			
Neurological injury	14	11 130	1917
Anemia	12	9965	856
Organ dysfunction or high illness severity^c^	25	7697	1863
Infection	13	7587	1994
Mechanical ventilation	14	7281	1468
Kidney injury	14	7047	1545
Pain	14	6259	1349
Hypoxemia	12	5085	2103
Leukocytosis	7	3307	641
Fever or hypothermia	8	3181	999
Stroke^d^	8	2653	519
Respiratory disease^e^	6	2164	593
Liver dysfunction	6	2118	477
Hypotension	4	1557	347
Tachypnea	2	851	170
Stress, anxiety, or depression	2	754	86
High thyroid-stimulating hormone level	1	568	82
Dehydration	1	566	566
Urinary retention	1	314	86
Thrombocytopenia	2	240	126
Cardiac arrest or cardiogenic shock	1	212	12
Unsafe swallow (on admission)	1	82	23
Hyperoxia^f^	1	65	19
Metabolic abnormality			
Glucose level	3	6704	403
Albumin level	10	6260	1120
Electrolyte imbalance	3	2333	251
Metabolic acidosis	2	1618	247
Metabolic disturbance or disorder	3	1457	868
Sodium level	3	1065	193
Calcium level	1	818	90
Hyperamylasemia	1	818	90
Potassium level	2	365	72
Fluid level	2	270	52
Magnesium level	1	90	49
Pharmacology			
Benzodiazepine	11	5145	1078
Opioid	14	4.215	774
Sedative or analgesic	10	3295	1551
Neuroleptic	5	3032	688
Anticholinergic	4	2225	756
Multiple medications	4	1077	149
Patient-controlled analgesia	1	915	104
Statin discontinuation	1	763	588
Mannitol	1	618	131
Psychoactive drug	2	419	138
Steroid	2	391	125
Nicotine withdrawal	1	293	210
Acetylcholinesterase inhibitor	1	251	125
Nonsteroidal anti-inflammatory drug	1	80	36
Iatrogenic and environmental factor			
Urinary catheter	10	3812	1214
Physical restraint	11	2841	805
Longer length of stay	9	2724	461
ICU admission	4	1564	264
Neurosurgical drainage tube	1	800	157
Sleep disturbance	5	749	271
Fall	2	743	383
Bed or ward change	2	710	108
Immobilization	1	612	68
Gastric tube	1	320	92
Administration of therapy during night hours	1	203	35
Any iatrogenic event	1	196	35
Trauma^g^	5	1282	269
Biomarker			
High CRP level	13	4321	1163
High IL-6 level	7	1229	654
High neopterin level	5	672	274
High NT-proBNP level	1	635	73
High IL-8 level	3	604	435
High S100B level	3	575	541
High cortisol level	4	527	208
Low ubiquitin C-terminal hydrolase level	1	427	327
Low cerebral oxygen saturation	3	395	98
High micro-RNA-210 level	1	370	63
Low IGF-1 level	3	326	71
High TNF-a level	1	321	321
High IL-10 level	1	321	321
Higher CSF p-tau level	1	214	57
Change in exosomal a-synuclein	1	202	17
High procalcitonin level	1	149	30
Endothelial dysfunction	1	147	103
High CSF sTREM2 level	1	146	65
Burst suppression	1	141	20
High sTNFR11 level	1	138	107
Low MMP-9 level	1	138	107
Low protein C level	1	138	107
High plasminogen activator inhibitor level	1	134	94
High E-selectin level	1	134	94
High reactive hyperemia index level (endothelial activation)	1	134	94
High CSF t-τ level	1	129	70
Autoregulation function^h^	2	118	37
High IL-2 level	1	113	41
Altered energy metabolism and amino acid synthesis^i^	1	104	52
High Lp-PLA2	1	62	15
Neurotransmitter level			
High CSF or serum tryptophan	3	470	93
Low CSF or serum BuChE	1	447	51
Low CSF or serum AChE	1	447	51
High CSF or serum ChAT	1	447	51
High CSF or serum phenylalanine	2	373	93
Low plasma leptin	1	336	102
High serum homovanillic acid	1	125	58
Low serum acetylcholine	1	119	19
Low plasma esterase	1	101	37
High CSF or serum tyrosine	2	77	53
High CSF methionine	1	77	53
High CSF 5-HIAA	1	77	53

Abbreviations: AChE, acetylcholinesterase; BuChE, butyrylcholinesterase; ChAT, choline acetyltransferase; CRP, C-reactive protein; CSF, cerebrospinal fluid; ICU, intensive care unit; IGF-1, insulin-like growth factor-1; IL, interleukin; Lp-PLA2, lipoprotein-associated phospholipase; MMP-9, matrix metalloproteinase-9; NT-proBNP, N-terminal prohormone of brain natriuretic peptide; p-tau, phosphorylated tau; sTNFR11, soluble tumor necrosis factor receptor-1; sTREM2, soluble fragment of triggering receptor expressed on myeloid cells 2; S100B, S100 calcium binding protein B; TNF-a, tumor necrosis factor alpha; 5-HIAA, 5-hydoxyindoleacetic acid.

^a^ Emergent or urgent surgery, open surgery, invasive surgery, fixation, knee surgery, orthopedic surgery, spine and major joints arthroplasty, spine surgery, intrathoracic surgery, abdominal aneurysm surgery, combined coronary artery bypass graft and valve surgery, vascular surgery, osteosynthesis surgery, segmentectomy, lobectomy resection, or frontal approach craniotomy.

^b^ Combined intravenous and inhalational anesthesia or regional anesthesia.

^c^ Measured by Acute Physiology and Chronic Health Evaluation, multiple organ failure, or Sequential Organ Failure Assessment.

^d^ Intracerebral hemorrhage, neglect, apraxia, inability to lift both arms on admission, vision deficit, anterior circulation infarcts, left cortical infarct, and posterior circulation infarcts.

^e^ Respiratory disease (unspecified), respiratory acidosis, or noninvasive ventilation.

^f^ Measured as increased partial pressure of oxygen.

^g^ Severe trauma, fracture, or amputation.

^h^ Measured as an autoregulation index calculated from cerebral blood flow velocity response to a step change in blood pressure and by the transient hyperemic response ratio index.

^i^ Valine, leucine, and isoleucine synthesis; nicotinate and nicotinamide metabolism; pyrimidine metabolism; 1 carbon pool by folate; glycine, serine, and threonine metabolism; arginine biosynthesis; citrate cycle; cysteine and methionine metabolism; pentose phosphate pathway; glyoxylate and dicarboxylate metabolism; pyrimidine metabolism; alanine, aspartate, and glutamate metabolism; ascorbate and aldarate metabolism; or pyruvate metabolism.

**Table 4.** Precipitating Factors Associated With Delirium and Their Mechanisms

Precipitating factors	Putative pathophysiological mechanism
Duration of operation, anesthesia type and depth, sedative or analgesic, opioid, neuroleptic, benzodiazepine, psychoactive drug, acetylcholinesterase inhibitor, and anticholinergic	Exposure to neurologically active medication or substance
Intraoperative blood transfusion; intraoperative hemodynamics; postoperative complication, atrial fibrillation, or shock; intraoperative fluids; anemia; hypotension; and cardiac arrest or cardiogenic shock	Hypoperfusion
Neurological injury, stroke, and seizure^a^	Neuronal injury
Infection, leukocytosis, and meningoencephalitis^a^	Systemic inflammation and neuroinflammation
Mechanical ventilation, hypoxemia, respiratory disease, and tachypnea	Hypoxia or hypercarbia
Glucose abnormality	Glucose extremes
Kidney injury and liver dysfunction	Uremia, impaired clearance of neurologically active medications or substances. or both
Electrolyte imbalance; metabolic disturbance or disorder; sodium, calcium, potassium, or magnesium level; metabolic acidosis; or hyperamylasemia	Electrolyte disturbances
Pain, urinary retention, sleep disturbance, and administration of therapies during night hours	Pain and/or sleep or circadian rhythm disruption
Urinary catheter, physical restraint, gastric tube, and falls	Immobilization
Low albumin level, thiamine deficiency,^a^ niacin deficiency,^a^ and cobalamin deficiency^a^	Malnutrition/cofactor deficiency
Fever or hypothermia	Temperature extremes
Nicotine withdrawal and alcohol withdrawal^a^	Withdrawal from neurologically active medications/substances
Steroid	Hyperadrenergic state
Type of surgery, organ dysfunction or high illness severity, time to operation, number of surgeries, intensive care unit admission, longer length of stay, trauma, multiple medications, statin discontinuation, any iatrogenic event, and >3 bed changes	Likely epiphenomenal variables representing presence of one or more of the above mechanisms, association with one or more predisposing factors, or found due to confounding

^a^ Precipitating factors not listed in Table 3.

### Factors Associated With Decreased Risk of Delirium

There were 17 studies^20,21,22,23,98,105,135,165,175,184,234,240,297,307,315,323,327^ that identified factors associated with a decreased risk of delirium. The most robust protective factor was cognitive reserve, which is a complex construct indicating the ability of an individual to withstand changes or stresses on brain function. There were 3 studies,^20,21,22^ with 1487 participants, that identified some but not all markers of cognitive reserve as protective factors, including high vocabulary level or score and more frequent engagement in cognitive and social activities.^20,21,22^ Other protective factors identified in single, small studies included oral opioids (compared with patient-controlled analgesia)^297^; opioid prescription (eg, by reducing pain)^105^; environmental factors, including having a television or radio in the hospital room, number of hours mobilized, geriatric comanagement, and being in a single room in the intensive care unit^22,105,234,307,323^; use of vasopressors, sleep aids, regional anesthesia, or inotropic, antihypertensive, antianginal, and antiretroviral medications^98,135,165,307^; and an increase in measured brain tissue oxygenation, mean arterial pressure, or albumin levels.^165,240,315^ Increased level of amyloid-β 1-42 in cerebrospinal fluid was identified as a protective factor in 1 study.^23^ Because our systematic review was not designed to identify all studies examining factors associated with decreased risk of delirium, this list is not exhaustive.

## Discussion

We conducted a systematic review of the literature to identify predisposing and precipitating factors associated with delirium. Across 315 included studies^17,18,19,20,21,22,23,24,25,26,27,28,29,30,31,32,33,34,35,36,37,38,39,40,41,42,43,44,45,46,47,48,49,50,51,52,53,54,55,56,57,58,59,60,61,62,63,64,65,66,67,68,69,70,71,72,73,74,75,76,77,78,79,80,81,82,83,84,85,86,87,88,89,90,91,92,93,94,95,96,97,98,99,100,101,102,103,104,105,106,107,108,109,110,111,112,113,114,115,116,117,118,119,120,121,122,123,124,125,126,127,128,129,130,131,132,133,134,135,136,137,138,139,140,141,142,143,144,145,146,147,148,149,150,151,152,153,154,155,156,157,158,159,160,161,162,163,164,165,166,167,168,169,170,171,172,173,174,175,176,177,178,179,180,181,182,183,184,185,186,187,188,189,190,191,192,193,194,195,196,197,198,199,200,201,202,203,204,205,206,207,208,209,210,211,212,213,214,215,216,217,218,219,220,221,222,223,224,225,226,227,228,229,230,231,232,233,234,235,236,237,238,239,240,241,242,243,244,245,246,247,248,249,250,251,252,253,254,255,256,257,258,259,260,261,262,263,264,265,266,267,268,269,270,271,272,273,274,275,276,277,278,279,280,281,282,283,284,285,286,287,288,289,290,291,292,293,294,295,296,297,298,299,300,301,302,303,304,305,306,307,308,309,310,311,312,313,314,315,316,317,318,319,320,321,322,323,324,325,326,327,328,329,330,331^ with 101 144 patients (of whom 24 015 patients had delirium), we identified 33 predisposing and 112 precipitating factors associated with delirium. Unlike many recent systematic reviews of risk factors associated with delirium, ours was agnostic to setting and identified predisposing and precipitating factors using a methodology for distinguishing between them. This review provides an up-to-date and comprehensive library of delirium etiologies, which may be used to inform more precise study of delirium pathophysiology and its treatment. Our findings differ from those of prior reviews in that they reflect the delirium literature in its entirety, across all populations and settings.

Our results reinforce previously cited risk factors associated with delirium, including advanced age, dementia, cognitive impairment, frailty, history of delirium or other central nervous system disorders, cumulative comorbidities, alcohol use, depression, malnutrition, and functional, visual, or hearing impairment.^2,332,333^ We also identified several less commonly discussed predisposing factors, including cardiovascular disease, lower educational attainment, anemia, tobacco use, polypharmacy, diabetes, anxiety, pain, obstructive sleep apnea, chronic obstructive pulmonary disease, and chronic kidney disease.^24,25,26,27,28,29,30,31,32,33,34,35,36,37,38,39,40,41,42,43,44,45,46,47,48,49,50,51,52,53,54,55,56,57,58,59,334^ Our results also suggest that some associations were not consistent across settings, such as male sex being a risk factor associated with delirium in many studies but female sex being a risk factor associated with delirium in others.

There are some important precipitating factors associated with delirium that our study did not identify owing to their low likelihood of being identified in population-based studies. However, these are well-known factors associated with this delirium phenotype and often primary neurological disorders, such as meningitis, encephalitis (infectious and autoimmune, including paraneoplastic factors), hydrocephalus, seizure, alcohol and other substance withdrawal, and specific vitamin deficiencies, such as those of thiamine, niacin, and cobalamin.

The practice of classifying delirium by its psychomotor activity (hyperactive, hypoactive, or mixed level of activity) has offered little to advance research or clinical practice. For instance, activity level is not consistently associated with etiology, and psychomotor subtypes are seldom helpful in formulating interventions.^335,336,337,338^ The *DSM-5* includes 5 etiologic subtypes (substance intoxication, substance withdrawal, medication induced, due to another medical condition, and due to multiple etiologies), although these categories contain too much biological heterogeneity to inform clinical practice beyond treating the underlying cause.^339^ Additionally, prior reports have attempted to categorize delirium by clinical symptom, including with the use of factor analysis or by motor subtype, age group, or outcome, although such approaches have yet to yield a set of discrete physiological subtypes that inform interventions.^10,340,341,342,343,344,345,346^

Whereas the pathophysiology of delirium is poorly understood, the weight of the evidence implicates several, variously interrelated biological factors, including neurotransmitters, inflammation, physiological stressors, metabolic derangements, electrolyte disorders, and genetic factors, in disrupting neuronal networks by directly or indirectly interfering with neuronal and glial activity.^2,347,348^ Frequently implicated neurotransmitter systems include acetylcholine and melatonin deficiency, dopaminergic excess, norepinephrine or glutamate release, and alterations in serotonin, histamine, or γ-amino butyric acid levels; however, it is unlikely that any single pattern of neurotransmitter disturbances underlies all instances of delirium.^349^ Inflammation may cause delirium by way of stress-induced cytokines^350^ and microglial reactivity.^351^ Neuronal activity may be disrupted by diverse physiological stressors, including hypoxia, extremes in temperature and glucose level, metabolic and electrolyte derangement, seizure, and direct injury, such as that due to stroke or trauma. Effective delirium treatment will likely depend on identifying precise biological factors associated with each episode.

By providing a comprehensive list of predisposing and precipitating factors associated with delirium based on a systematic review, this study may provide a starting point from which to categorize delirium episodes by etiology. We provided an example of how precipitating factors associated with delirium identified in this systematic review may be grouped by putative primary pathophysiological mechanism. Many precipitating factors are associated with delirium via multiple mechanisms. For example, physical restraints may be associated with delirium through immobilization, sleep or circadian rhythm disruption, or induction of a hyperadrenergic state. Nevertheless, the framework illustrated in this study may be used to classify episodes of delirium by their primary putative pathophysiology to inform future studies and clinical trials so that unique patterns of pathophysiology are included among clinical management targets. In addition, many episodes of delirium are multifactorial, and this framework may help identify which multiple factors were associated with a given delirium episode. It is also likely that several precipitating factors identified in this review were epiphenomenal, meaning that they were associated with other factors through confounding by unmeasured or incompletely measured variables. It should be a research priority to differentiate epiphenomenal from causal mechanisms to guide the development of effective interventions.

This systematic review has many strengths. It followed the PRISMA guideline and involved an expansive literature search. Rigorous inclusion and exclusion criteria were used so that delirium was assessed prospectively by qualified clinicians or research personnel using reference standards. Predisposing and precipitating factors were listed only when identified in multivariable models. To our knowledge, this is the first systematic review to include the full breadth of the delirium literature, independent of setting, population, or etiology. We have provided a comprehensive description of predisposing and precipitating factors associated with delirium reported across studies.

### Limitations

We acknowledge several limitations of this review. Given the lack of consistent terminology in delirium research, we may have excluded studies despite the use of broad MeSH terms with the aim of capturing all studies of delirium and confusion. The heterogeneity of included studies limited our synthesis to narrative review; we do not provide quantitative measures of risk factors associated with delirium, although the number of studies and participants may provide some measure of consistency and strength of association. This approach was taken to investigate limits of current understanding and encourage hypothesis testing. Heterogeneity also precluded our ability to perform a meta-analysis. It is possible that some factors identified only in single studies would be eliminated in a meta-analysis of larger cohorts; these may reflect statistical chance rather than true association. For example, White race was identified as a predisposing factor associated with delirium in a single study^17^ in which 51 of 309 participants (16.5%) were members of racial minority groups, 77.3% of participants experienced delirium, and there was a high risk of overfitting given that 10 variables were included in the multivariable model.^17^ Because race was not identified as a predisposing factor associated with delirium in 2 larger studies^18,19^ in this review, it is more likely that race was not associated with delirium risk. Conversely, other factors identified in single studies may be insufficiently investigated. For example, a single study^18^ identified non-English language as a predisposing factor. This may be more likely to represent an association and an area in need of further study given that 138 studies in this review (43.8%) excluded participants due to a language barrier (participants were excluded if they did not speak the same language that delirium assessors used). This review included biomarkers when identified by studies that met inclusion and exclusion criteria and is not an exhaustive review of biomarkers for delirium. Additionally, this literature search did not include research on the association of the COVID-19 virus and pandemic with delirium.

## Conclusions

This systematic review found a tremendous range of predisposing and precipitating factors associated with delirium. The best explanation for the physiological heterogeneity represented across studies may be that delirium cannot be restricted to a singular physiological event or sequence of events. Delirium is a convergent clinical syndrome, and discrete yet often interacting pathophysiological processes are associated with this syndrome, each of which warrants targeted interventions. These findings argue against a one-size-fits-all approach to understanding, identifying, and treating delirium; they encourage a reappraisal of delirium from the perspective of its multiple physiological pathways. Current management includes treating the underlying cause, managing psychiatric symptoms, and promoting procognitive factors. Multicomponent, nonpharmacological approaches are associated with decreased delirium incidence but with prevention of a minority of incidences and have not been shown to be associated with a reverse in delirium once it has developed.^352,353^ Whereas we advocate for multicomponent approaches to delirium, we would go a step further and advocate for a reconceptualization of delirium in terms of putative endotypes. That is, we emphasize the need to target individual, specific physiological pathways. Predisposing and precipitating factors identified here implicate a range of pathophysiological types and strongly imply that delirium research and clinical management should not only specify factors associated with delirium, but also consider underlying pathophysiology. This systematic review provides a comprehensive library of predisposing and precipitating factors associated with delirium, which may be used to inform the study of delirium pathophysiology and its treatment.

## Notes

Supplement 1. **eTable.** Participants and Quality Assessment of 315 Included Studies **eFigure.** Study Flowchart

Supplement 2.
Data Sharing Statement
