# Supplementary material for: Predisposing and Precipitating Factors Associated With Delirium: A Systematic Review
Source: JAMA Netw Open. 2023 Jan 6;6(1):e2249950. doi: 10.1001/jamanetworkopen.2022.49950 (PMC9856673; doi:10.1001/jamanetworkopen.2022.49950)
Supplement: Supplement 1. — eTable. Participants and Quality Assessment of 315 Included Studies eFigure. Study Flowchart [file jamanetwopen-e2249950-s001.pdf]

Supplemental Online Content

Ormseth CH, LaHue SC, Oldham MA, Josephson SA, Whitaker E, Douglas VC. Predisposing and precipitating factors associated with delirium: a systematic review. *JAMA Netw Open*. 2023;6(1):e2249950 doi:10.1001/jamanetworkopen.2022.49950

eTable. Participants and Quality Assessment of 315 Included Studies
eFigure. Study Flowchart

This supplemental material has been provided by the authors to give readers additional information about their work.

© 2023 Ormseth CH et al. *JAMA Network Open.*

eTable. Participants and Quality Assessment of 315 Included Studies

Author, Year	Total Participants (N)	Participants with Delirium (N)	Newcastle-Ottawa Scale (9 points maximum)
Aldemir 2001[1]	818	90	9
Angles 2008[2]	69	41	8
Ansaloni 2010[3]	351	47	9
Bakker 2012[4]	201	63	9
Banach 2008[5]	260	30	8
Banjongrewadee 2020[6]	429	22	9
Behrends 2013[7]	472	137	9
Beishuizen 2017[8]	385	127	8
Beland 2021[9]	612	68	9
Bell 2021[10]	127	69	8
Benoit 2005[11]	102	34	8
Bisschop 2011[12]	143	70	7
Bo 2016[13]	330	52	9
Bohner 2003[14]	153	60	8
Brouquet 2010[15]	118	28	7
Bryson 2011[16]	87	30	8
Budenas 2018[17]	522	22	8
Burkhart 2010[18]	113	35	8
Caeiro 2004[19]	218	29	9
Caeiro 2004[20,a]	74	22	9
Cai 2020[21]	635	73	9
Caldas 2019[22]	67	17	8
Carrasco 2014[23]	374	25	9
Cavallari 2015[24]	146	32	9
Cavallari 2016[25]	136	29	9
Cerejeira 2011[26]	101	37	9
Chaiwat 2019[27]	250	61	8
Chan 2021[28]	199	73	9
Chen 2017[29]	212	35	9
Chen 2020[30]	370	63	9
Chen 2021[31]	383	66	9
Cheng 2019[32]	119	28	8
Chou 2019[33]	461	37	9
Chouet 2020[34]	240	6	9
Chu 2016[35]	544	52	9

© 2023 Ormseth CH et al. *JAMA Network Open.*

Author, Year	Total Participants (N)	Participants with Delirium (N)	Newcastle-Ottawa Scale (9 points maximum)
Cole 2011[36]	104	68	6
Colkesen 2013[37]	52	25	9
Contin 2005[38]	236	52	8
Cunningham 2017[39]	315	40	8
Cunningham 2019[40,a]	282	40	8
Dahl 2010[41]	178	18	8
Daoust 2020[42]	338	41	8
Day 2021[43]	1182	172	7
De la Varga-Martinez[44]	345	58	8
De Rooij 2007[45]	185	64	8
Detroyer 2008[46]	104	27	9
Dillon 2017[47]	150	75	9
Douglas 2013[48]	209	25	9
Du Plooy 2020[49]	808	99	7
Duceppe 2019[50]	150	58	8
Dworkin 2016[51]	76	10	9
Edlund 1999[52]	54	15	8
Edlund 2001[53]	101	49	8
Edlund 2006[54]	400	125	8
Egberts 2015[55]	86	23	8
Egberts 2019[56]	210	80	8
Eide 2015[57]	143	76	8
Ely 2007[58]	53	47	8
Fan 2019[59]	336	68	8
Fann 2002[60,a]	90	49	8
Fann 2011[61]	90	45	8
Feast 2018[62]	227	26	9
Feng 2021[63]	51	20	8
Fick 2013[64]	139	44	8
Fisher 1995[65]	80	14	8
Flink 2012[66]	106	27	9
Fong 2015[67]	300	82	9
Fortini 2014[68]	560	63	8
Foy 1995[69]	418	21	8
Franco 2010[70]	291	34	9
Freter 2005[71]	100	24	7
Freter 2015[72]	283	163	8

© 2023 Ormseth CH et al. *JAMA Network Open.*

Author, Year	Total Participants (N)	Participants with Delirium (N)	Newcastle-Ottawa Scale (9 points maximum)
Galanakis 2001[73]	105	25	9
Girard 2012[74]	138	107	8
Girard 2018[75]	1040	740	8
Giroux 2018[76]	335	40	8
Goldenberg 2006[77]	77	37	9
Goudzwaard 2020[78]	543	75	8
Greene 2009[79]	100	16	8
Gu 2021[80]	618	131	8
Gual 2018[81]	909	352	7
Guo 2016[82]	572	120	8
Guo 2017[83]	385	56	9
Hall 2016[84]	139	64	8
Hatta 2019[85]	526	93	9
Hayhurst 2020[86]	427	327	8
He 2020[87]	780	182	9
Hein 2014[88]	410	102	8
Heng 2016[89]	739	83	6
Henjum 2018[90]	146	65	8
Hirsch 2015[91]	540	178	8
Hsieh 2015[92]	532	241	8
Huang 2021[93]	800	157	8
Hughes 2013[94]	147	103	8
Hughes 2016[95]	134	94	8
Humbert 2021[96]	93	51	8
Hwang 2018[97]	162	21	9
Iamaroon 2020[98]	249	29	8
Idland 2017[99]	129	70	8
Inouye 1993[100]	107	27	9
Inouye 1996[101]	196	35	9
Inouye 2007[102]	491	106	9
Jankowski 2011[103]	418	42	9
Jones 2006[104]	952	169	9
Joosten 2006[105]	190	34	8
Joosten 2014[106]	220	24	8
Juliebo 2009[107]	364	168	8
Kagansky 2004[108]	102	12	8
Kalisvaart 2006[109]	603	74	9

© 2023 Ormseth CH et al. *JAMA Network Open.*

Author, Year	Total Participants (N)	Participants with Delirium (N)	Newcastle-Ottawa Scale (9 points maximum)
Kang 2020[110]	138	25	7
Kanova 2017[111]	284	74	8
Kazmierski 2010[112]	563	92	9
Kazmierski 2013[113,a]	113	41	9
Kazmierski 2014[114,a]	113	41	9
Kazmierski 2014[115,a]	113	41	9
Kazmierski 2014[116,a]	102	32	9
Kennedy 2014[117]	676	63	7
Khan 2016[118]	1589	150	8
Khan 2020[119]	321	321	8
Khan 2020[120]	2742	452	9
Kim 2016[121]	561	112	9
Kim 2017[122]	194	37	9
Kim 2018[123]	104	15	9
Kim 2020[124]	175	107	8
Kong 2021[125]	98	30	8
Korevaar 2005[126]	126	36	8
Kosar 2014[127]	459	106	9
Koskderelioglu 2017[128]	109	20	9
Kostalova 2012[129]	100	43	8
Krzych 2013[130]	5781	236	8
Kumar 2017[131]	120	21	9
Kupiec 2020[132]	149	30	8
Kwizera 2015[133]	160	81	8
Lai 2012[134]	300	300	7
Lee 2011[135]	425	149	9
Lee 2011[136]	232	70	8
Lee 2019[137]	132	41	9
Leung 2005[138]	219	101	8
Leung 2007[139]	203	29	9
Leung 2011[140]	63	16	9
Leung 2013[141]	581	234	9
Levkoff 1992[142]	325	125	8
Lewis 2017[143]	494	89	8
Li 2017[144]	336	102	9
Li 2020[145]	115	76	9
Liang 2015[146]	461	37	9

© 2023 Ormseth CH et al. *JAMA Network Open.*

Author, Year	Total Participants (N)	Participants with Delirium (N)	Newcastle-Ottawa Scale (9 points maximum)
Lin 2020[147]	301	106	8
Lin 2020[148]	447	51	8
Lin 2021[149]	214	57	8
Limpawattana 2016[150]	99	44	8
Lindroth 2019[151]	97	31	8
Litaker 2001[152]	500	57	9
Ma 2020[153]	119	19	8
Ma 2021[154]	325	64	8
Mahanna-Gabrielli 2020[155]	178	45	8
Marcantonio 1994[156]	245	91	8
Marcantonio 1998[157]	1341	117	8
Martin 2000[158]	156	28	9
Martinez 2012[159]	397	52	7
Matsuda 2020[160]	566	566	7
Matsuo 2017[161]	207	35	9
Mazzola 2017[162]	415	124	9
McAlpine 2008[163]	103	18	7
McAvay 2007[164]	416	36	9
McCusker 2011[165]	279	82	8
McManus 2009[166]	82	23	7
McNicoll 2003[167]	118	83	8
McPherson 2013[168]	200	53	8
Meziere 2013[169]	52	7	7
Miao 2018[170]	112	49	8
Miu 2013[171]	314	86	8
Miu 2016[172]	261	89	8
Moorey 2016[173]	251	125	8
Morandi 2011[174]	110	72	8
Morandi 2013[175]	120	37	8
Morandi 2014[176]	763	588	8
Mori 2016[177]	149	69	8
Morrison 2003[178]	541	87	9
Mu 2010[179]	243	123	8
Muangpaisan 2015[180]	80	36	8
Neerland 2017[181]	696	124	9
Nekrosius 2019[182]	89	17	8

© 2023 Ormseth CH et al. *JAMA Network Open.*

Author, Year	Total Participants (N)	Participants with Delirium (N)	Newcastle-Ottawa Scale (9 points maximum)
Neufeld 2013[183]	91	41	8
Ngo 2017[184]	78	39	9
Nie 2012[185]	123	16	8
Noriega 2015[186]	203	35	9
Norkiene 2007[187]	1367	42	8
O'Regan 2018[188]	191	61	9
Oh 2016[189]	431	147	9
Ojagbemi 2017[190]	111	33	7
Oldham 2019[191]	131	21	9
Oliveira 2018[192]	173	59	9
Osse 2012[193]	125	58	9
Otomo 2013[194]	153	16	8
Pandharipande 2006[195]	198	Not reported	8
Pandharipande 2009[196]	97	Not reported	8
Park 2016[197]	293	210	8
Pasinska 2018[198]	750	203	8
Patrono 2020[199]	306	41	7
Pedemonte 2020[200]	141	20	9
Pendlebury 2015[201]	503	101	9
Peng 2019[202]	272	55	8
Perez-Ros 2019[203]	443	83	8
Pioli 2019[204]	939	292	9
Pipanmekaporn 2021[205]	429	23	9
Pisani 2015[206]	309	239	8
Pollmann 2021[207]	197	80	9
Pompei 1994[208]	432	64	8
Qi 2020[209]	326	68	9
Qu 2018[210]	261	38	7
Racine 2017[211]	145	32	8
Radinovic 2015[212]	270	143	9
Radinovic 2019[213]	277	148	9
Ranhoff 2006[214]	401	117	9
Rao 2020[215]	187	67	8
Ren 2020[216]	206	12	8
Ritchie 2014[217]	710	87	7
Rizzi 2015[218]	239	35	8

© 2023 Ormseth CH et al. *JAMA Network Open.*

Author, Year	Total Participants (N)	Participants with Delirium (N)	Newcastle-Ottawa Scale (9 points maximum)
Robinson 2009[219]	144	64	8
Roggenbach 2014[220]	92	44	8
Rudolph 2006[221]	80	40	9
Rudolph 2007[222]	1161	99	8
Rudolph 2009[223]	68	33	9
Saczynski 2014[224]	566	135	8
Sanchez-Hurtado 2018[225]	109	25	8
Santos 2004[226]	220	74	8
Schoen 2011[227]	231	62	8
Schor 1992[228]	291	91	9
Schreiber 2014[229]	330	99	9
Serafim 2012[230]	465	43	8
Seymour 2012[231]	140	Not reported	8
Shen 2016[232]	140	36	9
Sheng 2006[233]	156	39	8
Shim 2015[234]	631	195	8
Shin 2016[235]	78	40	9
Shioiri 2010[236]	116	19	8
Shioiri 2016[237,a]	116	19	8
Sieber 2011[238]	236	60	9
Siew 2017[239]	466	350	8
Singler 2014[240]	133	19	7
Slatore 2012[241]	105	54	7
Slor 2011[242]	526	60	9
Slor 2019[243]	121	41	8
Smulter 2013[244]	142	78	7
Smulter 2018[245,a]	142	78	7
Soehle 2015[246]	81	26	8
Soh 2020[247]	113	16	9
Sosa 2018[248]	178	49	9
Srinonprasert 2011[249]	225	110	8
Stubljar 2019[250]	103	7	7
Styra 2019[251]	173	20	8
Susano 2020[252]	219	55	8
Tahir 2018[253]	381	70	7
Taipale 2012[254]	122	27	8
Tan 2008[255]	53	12	7

© 2023 Ormseth CH et al. *JAMA Network Open.*

Author, Year	Total Participants (N)	Participants with Delirium (N)	Newcastle-Ottawa Scale (9 points maximum)
Tao 2019[256]	507	112	8
Thillainadesan 2021[257]	150	15	8
Thisayakorn 2021[258]	65	19	8
Theologou 2018[259]	179	20	8
Tiwary 2021[260]	80	13	7
Todd 2017[261]	101	27	8
Tong 2020[262]	154	34	8
Tow 2016[263]	142	45	8
Tripp 2021[264]	104	52	9
Tsuruta 2010[265]	106	21	8
Tully 2010[266]	158	49	8
Uchida 2015[267]	61	26	8
Uguz 2010[268]	212	12	8
Van der Mast 2000[269]	296	40	8
Van der Wulp 2019[270]	703	116	8
VanGrootven 2016[271]	86	24	9
vanMunster 2008[272]	98	50	8
vanMunster 2010[273]	412	126	8
vanMunster 2010[274]	720	264	8
vanMunster 2010[275]	120	62	8
vanMunster 2012[276]	142	72	8
Vasunilashorn 2017[277]	560	134	8
Vasunilashorn 2019[278]	547	127	8
Vaurio 2006[279]	333	144	8
Veliz-Reissmuller 2007[280]	107	25	8
Verloo 2016[281]	114	22	8
Villalpando-Berumen 2003[282]	667	80	9
Vondeling 2020[283]	1090	513	9
Wada 2019[284]	91	29	8
Wan 2020[285]	62	15	9
Wang 2018[286]	128	54	8
Wang 2020[287]	800	157	8
Wang 2020[288]	310	118	8
Wang 2021[289]	319	85	9
Wang 2021[290]	1266	317	8

© 2023 Ormseth CH et al. *JAMA Network Open.*

Author, Year	Total Participants (N)	Participants with Delirium (N)	Newcastle-Ottawa Scale (9 points maximum)
Watne 2014[291]	151	72	8
Watne 2016[292]	77	53	8
Wesselink 2015[293]	734	99	8
Wilson 2005[294]	100	12	9
Witlox 2011[295]	76	30	8
Witlox 2020[296]	75	27	9
Wood 2017[297]	103	19	8
Wu 2021[298]	228	57	7
Xie 2014[299]	153	31	8
Xing 2019[300]	320	92	8
Xu 2021[301]	568	82	8
Yam 2018[302]	575	91	8
Yang 2008[303]	779	Not reported	8
Yen 2016[304]	106	22	9
Yoshimura 2004[305]	100	17	8
Yuan 2020[306]	202	17	9
Zaal 2013[307]	130	62	8
Zhang 2018[308]	700	111	8
Zhang 2020[309]	288	49	7
Zhang 2021[310]	915	339	8
Zhang 2021[311]	1083	196	8
Zhao 2020[312]	288	49	9
Zhao 2021[313]	740	101	9
Zipprich 2020[314]	611	64	7
Zrour 2020[315]	230	59	7

[a] Participants were also included in another study in this table and therefore were not included in the sum of total participants.

References

1. Aldemir M, Özen S, Kara IH, Sir A, Baç B. Predisposing factors for delirium in the surgical intensive care unit. Article. *Critical Care*. 2001;5(5):265-270. doi:10.1186/cc1044
2. Angles EM, Robinson TN, Biffl WL, et al. Risk factors for delirium after major trauma. *American journal of surgery*. Dec 2008;196(6):864-9; discussion 869-70. doi:10.1016/j.amjsurg.2008.07.037
3. Ansaloni L, Catena F, Chattat R, et al. Risk factors and incidence of postoperative delirium in elderly patients after elective and emergency surgery. *The British journal of surgery*. Feb 2010;97(2):273-80. doi:10.1002/bjs.6843

© 2023 Ormseth CH et al. *JAMA Network Open.*

4. Bakker RC, Osse RJ, Tulen JH, Kappetein AP, Bogers AJ. Preoperative and operative predictors of delirium after cardiac surgery in elderly patients. *European journal of cardio-thoracic surgery : official journal of the European Association for Cardio-thoracic Surgery*. Mar 2012;41(3):544-9. doi:10.1093/ejcts/ezr031
5. Banach M, Kazmierski J, Kowman M, et al. Atrial fibrillation as a nonpsychiatric predictor of delirium after cardiac surgery: a pilot study. *Medical science monitor : international medical journal of experimental and clinical research*. May 2008;14(5):Cr286-291.
6. Banjongrewadee M, Wongpakaran N, Wongpakaran T, Pipanmekaporn T, Punjasawadwong Y, Mueankwan S. Role of perceived stress in postoperative delirium: An investigation among elderly patients. *Aging & Mental Health*. 2020/01//
2021-07-22</p> 2020;24(1):148-154. doi:10.1080/13607863.2018.1523881
7. Behrends M, DePalma G, Sands L, Leung J. Association between intraoperative blood transfusions and early postoperative delirium in older adults. *Journal of the American Geriatrics Society*. Mar 2013;61(3):365-70. doi:10.1111/jgs.12143
8. Beishuizen SJ, Scholtens RM, van Munster BC, de Rooij SE. Unraveling the Relationship Between Delirium, Brain Damage, and Subsequent Cognitive Decline in a Cohort of Individuals Undergoing Surgery for Hip Fracture. *Journal of the American Geriatrics Society*. Jan 2017;65(1):130-136. doi:10.1111/jgs.14470
9. Beland E, Nadeau A, Carmichael PH, et al. Predictors of delirium in older patients at the emergency department: a prospective multicentre derivation study. *CANADIAN JOURNAL OF EMERGENCY MEDICINE*. 2021/05//undefined 2021;23(3):330-336. doi:10.1007/s43678-020-00004-8
10. Bell JJ, Pulle RC, Lee HB, Ferrier R, Crouch A, Whitehouse SL. Diagnosis of overweight or obese malnutrition spells DOOM for hip fracture patients: A prospective audit. *CLINICAL NUTRITION*. 2021/04//undefined 2021;40(4):1905-1910. doi:10.1016/j.clnu.2020.09.003
11. Benoit AG, Campbell BI, Tanner JR, et al. Risk factors and prevalence of perioperative cognitive dysfunction in abdominal aneurysm patients. *Journal of vascular surgery*. Nov 2005;42(5):884-90. doi:10.1016/j.jvs.2005.07.032
12. Bisschop PH, de Rooij SE, Zwinderman AH, van Oosten HE, van Munster BC. Cortisol, insulin, and glucose and the risk of delirium in older adults with hip fracture. *Journal of the American Geriatrics Society*. Sep 2011;59(9):1692-6. doi:10.1111/j.1532-5415.2011.03575.x
13. Bo M, Bonetto M, Bottignole G, et al. Length of Stay in the Emergency Department and Occurrence of Delirium in Older Medical Patients. *Journal of the American Geriatrics Society*. May 2016;64(5):1114-9. doi:10.1111/jgs.14103
14. Bohner H, Hummel TC, Habel U, et al. Predicting delirium after vascular surgery: a model based on pre- and intraoperative data. *Annals of surgery*. Jul 2003;238(1):149-56. doi:10.1097/01.sla.0000077920.38307.5f
15. Brouquet A, Cudennec T, Benoist S, et al. Impaired mobility, ASA status and administration of tramadol are risk factors for postoperative delirium in patients aged 75 years or more after major abdominal surgery. *Annals of surgery*. Apr 2010;251(4):759-65. doi:10.1097/SLA.0b013e3181c1cfc9
16. Bryson GL, Wyand A, Wozny D, Rees L, Taljaard M, Nathan H. A prospective cohort study evaluating associations among delirium, postoperative cognitive dysfunction, and

© 2023 Ormseth CH et al. *JAMA Network Open.*

apolipoprotein E genotype following open aortic repair. *Canadian journal of anaesthesia = Journal canadien d'anesthesie*. Mar 2011;58(3):246-55. doi:10.1007/s12630-010-9446-6
17. Budėnas A, Tamašauskas Š, Šliaužys A, et al. Incidence and clinical significance of postoperative delirium after brain tumor surgery. Article. *Acta neurochirurgica*. 2018;160(12):2327-2337. doi:10.1007/s00701-018-3718-2
18. Burkhart CS, Dell-Kuster S, Gamberini M, et al. Modifiable and nonmodifiable risk factors for postoperative delirium after cardiac surgery with cardiopulmonary bypass. *Journal of cardiothoracic and vascular anesthesia*. Aug 2010;24(4):555-9. doi:10.1053/j.jvca.2010.01.003
19. Caeiro L, Ferro JM, Albuquerque R, Figueira ML. Delirium in the first days of acute stroke. *Journal of neurology*. Feb 2004;251(2):171-8. doi:10.1007/s00415-004-0294-6
20. Caeiro L, Ferro JM, Claro MI, Coelho J, Albuquerque R, Figueira ML. Delirium in acute stroke: a preliminary study of the role of anticholinergic medications. *European Journal of Neurology*. Oct 2004;11(10):699-704. doi:10.1111/j.1468-1331.2004.00897.x
21. Cai SN, Latour JM, Lin Y, et al. Preoperative cardiac function parameters as valuable predictors for nurses to recognise delirium after cardiac surgery: a prospective cohort study. *EUROPEAN JOURNAL OF CARDIOVASCULAR NURSING*. 2020/04//undefined 2020;19(4):310-319. doi:10.1177/1474515119886155
22. Caldas JR, Panerai RB, Bor-Seng-Shu E, et al. Dynamic cerebral autoregulation: A marker of post-operative delirium? *Clinical Neurophysiology*. Jan 2019;130(1):101-108. doi:10.1016/j.clinph.2018.11.008
23. Carrasco M, Villarroel L, Calderon J, Martinez G, Andrade M, Gonzalez M. Development and validation of a clinical predictive model for delirium in hospitalized older people. *Revista medica de Chile*. Jul 2014;142(7):826-832. doi:10.4067/s0034-98872014000700002
24. Cavallari M, Hshieh TT, Guttmann CRG, et al. Brain atrophy and white-matter hyperintensities are not significantly associated with incidence and severity of postoperative delirium in older persons without dementia. *Neurobiology of aging*. Jun 2015;36(6):2122-2129. doi:10.1016/j.neurobiolaging.2015.02.024
25. Cavallari M, Dai W, Guttmann CRG, et al. Neural substrates of vulnerability to postsurgical delirium as revealed by presurgical diffusion MRI. *Brain: A Journal of Neurology*. 2016 Apr 01
2017-09-25 2016;139(4):1282-1294. doi:http://dx.doi.org/10.1093/brain/aww010
26. Cerejeira J, Batista P, Nogueira V, Firmino H, Vaz-Serra A, Mukaetova-Ladinska EB. Low preoperative plasma cholinesterase activity as a risk marker of postoperative delirium in elderly patients. *Age and ageing*. Sep 2011;40(5):621-6. doi:10.1093/ageing/afr053
27. Chaiwat O, Chanidnuan M, Pancharoen W, et al. Postoperative delirium in critically ill surgical patients: Incidence, risk factors, and predictive scores. Article. *BMC anesthesiology*. 2019;19(1)doi:10.1186/s12871-019-0694-x
28. CK C, FE S, K B, et al. - Association of Depressive Symptoms With Postoperative Delirium and CSF Biomarkers. - *Am J Geriatr Psychiatry 2021 Dec;29(12):1212-1221 doi:*. (- 1545-7214 (Electronic)):T - ppublish.
29. Chen W, Ke X, Wang X, et al. Prevalence and risk factors for postoperative delirium in total joint arthroplasty patients: A prospective study. *General hospital psychiatry*. May 2017;46:55-61. doi:10.1016/j.genhosppsych.2017.03.008

© 2023 Ormseth CH et al. *JAMA Network Open.*

30. Chen Y, Zheng J, Chen J. Preoperative Circulating MiR-210, a Risk Factor for Postoperative Delirium Among Elderly Patients with Gastric Cancer Undergoing Curative Resection. *Current pharmaceutical design*. 2020 2020;26(40):5213-5219. doi:10.2174/1381612826666200617163857
31. Chen Y, Qin J. Modified Frailty Index Independently Predicts Postoperative Delirium and Delayed Neurocognitive Recovery After Elective Total Joint Arthroplasty. *The Journal of arthroplasty*. 2021/02//undefined 2021;36(2):449-453. doi:10.1016/j.arth.2020.07.074
32. Cheng Q, Li L, Yang M, et al. Moderate hypercapnia may not contribute to postoperative delirium in patients undergoing bronchoscopic intervention. *Medicine*. May 2019;98(22):e15906. doi:10.1097/md.0000000000015906
33. Chou MY, Wang YC, Peng LN, et al. Intraoperative blood transfusion predicts postoperative delirium among older patients undergoing elective orthopedic surgery: A prospective cohort study. *International journal of geriatric psychiatry*. Jun 2019;34(6):881-888. doi:10.1002/gps.5086
34. Chouet J, Sacco G, Karras SN, Llewellyn DJ, Sanchez-Rodriguez D, Annweiler C. Vitamin D and Delirium in Older Adults: A Case-Control Study in Geriatric Acute Care Unit. *FRONTIERS IN NEUROLOGY*. 2020/09/18/ 2020;11doi:10.3389/fneur.2020.01034
35. Chu C-S, Liang C-K, Chou M-Y, et al. Short-Form Mini Nutritional Assessment as a useful method of predicting the development of postoperative delirium in elderly patients undergoing orthopedic surgery. *General hospital psychiatry*. Jan 2016 - Feb
Jan 2016 - Feb 2016
2018-01-16 2016;38:15-20. doi:http://dx.doi.org/10.1016/j.genhosppsych.2015.08.006
36. Cole MG, McCusker J, Voyer P, et al. Subsyndromal delirium in older long-term care residents: incidence, risk factors, and outcomes. *Journal of the American Geriatrics Society*. Oct 2011;59(10):1829-36. doi:10.1111/j.1532-5415.2011.03595.x
37. Colkesen Y, Giray S, Ozenli Y, Sezgin N, Coskun I. Relation of serum cortisol to delirium occurring after acute coronary syndromes. *American Journal of Emergency Medicine*. Jan 2013;31(1):161-165. doi:10.1016/j.ajem.2012.07.001
38. Contin AM, Perez-Jara J, Alonso-Contin A, Enguix A, Ramos F. Postoperative delirium after elective orthopedic surgery. *International journal of geriatric psychiatry*. Jun 2005;20(6):595-7. doi:10.1002/gps.1335
39. Cunningham EL, Mawhinney T, Beverland D, et al. Observational cohort study examining apolipoprotein E status and preoperative neuropsychological performance as predictors of post-operative delirium in an older elective arthroplasty population. *Age and ageing*. Sep 1 2017;46(5):779-786. doi:10.1093/ageing/afx042
40. Cunningham EL, McGuinness B, McAuley DF, et al. CSF Beta-amyloid 1-42 Concentration Predicts Delirium Following Elective Arthroplasty Surgery in an Observational Cohort Study. Article. *Annals of surgery*. 2019;269(6):1200-1205. doi:10.1097/SLA.0000000000002684
41. Dahl MH, Ronning OM, Thommessen B. Delirium in acute stroke--prevalence and risk factors. *Acta neurologica Scandinavica Supplementum*. 2010;(190):39-43. doi:10.1111/j.1600-0404.2010.01374.x
42. Daoust R, Paquet J, Boucher V, Pelletier M, Gouin E, Emond M. Relationship Between Pain, Opioid Treatment, and Delirium in Older Emergency Department Patients. *ACADEMIC EMERGENCY MEDICINE*. 2020/08//undefined 2020;27(8):708-716. doi:10.1111/acem.14033

© 2023 Ormseth CH et al. *JAMA Network Open.*

43. Day C, Manning K, Abdullah F, et al. Delirium in HIV-infected patients admitted to acute medical wards post universal access to antiretrovirals in South Africa. *South African medical journal = Suid-Afrikaanse tydskrif vir geneeskunde*. 2021/10/05/ 2021;111(10):974-980. doi:10.7196/SAMJ.2021.v111i10.15628
44. de la Varga-Martinez O, Gomez-Pesquera E, Munoz-Moreno MF, et al. Development and validation of a delirium risk prediction preoperative model for cardiac surgery patients (DELIPRECAS): An observational multicentre study. *JOURNAL OF CLINICAL ANESTHESIA*. 2021/05//undefined 2021;69doi:10.1016/j.jclinane.2020.110158
45. de Rooij SE, van Munster BC, Korevaar JC, Levi M. Cytokines and acute phase response in delirium. *Journal of psychosomatic research*. May 2007;62(5):521-5. doi:10.1016/j.jpsychores.2006.11.013
46. Detroyer E, Dobbels F, Verfaillie E, Meyfroidt G, Sergeant P, Milisen K. Is Preoperative Anxiety and Depression Associated with Onset of Delirium After Cardiac Surgery in Older Patients? A Prospective Cohort Study. *Journal of the American Geriatrics Society*. Dec 2008;56(12):2278-2284. doi:10.1111/j.1532-5415.2008.02013.x
47. Dillon ST, Vasunilashorn SM, Ngo L, et al. Higher C-Reactive Protein Levels Predict Postoperative Delirium in Older Patients Undergoing Major Elective Surgery: A Longitudinal Nested Case-Control Study. *Biol Psychiatry*. Jan 15 2017;81(2):145-153. doi:10.1016/j.biopsych.2016.03.2098
48. Douglas VC, Hessler CS, Dhaliwal G, et al. The AWOL tool: derivation and validation of a delirium prediction rule. *Journal of hospital medicine*. Sep 2013;8(9):493-9. doi:10.1002/jhm.2062
49. Du Plooy N, Day C, Manning K, et al. Prevalence and outcome of delirium among acute general medical inpatients in Cape Town, South Africa. *South African medical journal = Suid-Afrikaanse tydskrif vir geneeskunde*. 2020/05/29/ 2020;110(6):519-524. doi:10.7196/SAMJ.2020.v110i6.14363
50. Duceppe MA, Williamson DR, Elliott A, et al. Modifiable Risk Factors for Delirium in Critically Ill Trauma Patients: A Multicenter Prospective Study. Article. *Journal of intensive care medicine*. 2019;34(4):330-336. doi:10.1177/0885066617698646
51. Dworkin A, Lee DS, An AR, Goodlin SJ. A Simple Tool to Predict Development of Delirium After Elective Surgery. *Journal of the American Geriatrics Society*. Nov 2016;64(11):e149-e153. doi:10.1111/jgs.14428
52. Edlund A, Lundstrom M, Lundstrom G, Hedqvist B, Gustafson Y. Clinical profile of delirium in patients treated for femoral neck fractures. *Dementia and geriatric cognitive disorders*. Sep-Oct 1999;10(5):325-9. doi:10.1159/000017163
53. Edlund A, Lundstrom M, Brannstrom B, Bucht G, Gustafson Y. Delirium before and after operation for femoral neck fracture. *Journal of the American Geriatrics Society*. Oct 2001;49(10):1335-40. doi:10.1046/j.1532-5415.2001.49261.x
54. Edlund A, Lundstrom M, Karlsson S, Brannstrom B, Bucht G, Gustafson Y. Delirium in older patients admitted to general internal medicine. *Journal of geriatric psychiatry and neurology*. Jun 2006;19(2):83-90. doi:10.1177/0891988706286509
55. Egberts A, Wijnbeld EH, Fekkes D, et al. Neopterin: a potential biomarker for delirium in elderly patients. *Dementia and geriatric cognitive disorders*. 2015;39(1-2):116-24. doi:10.1159/000366410

© 2023 Ormseth CH et al. *JAMA Network Open.*

56. Egberts A, Osse RJ, Fekkes D, Tulen JHM, van der Cammen TJM, Mattace-Raso FUS. Differences in potential biomarkers of delirium between acutely ill medical and elective cardiac surgery patients. Article. *Clinical interventions in aging*. 2019;14:271-281. doi:10.2147/CIA.S193605
57. Eide LS, Ranhoff AH, Fridlund B, et al. Comparison of frequency, risk factors, and time course of postoperative delirium in octogenarians after transcatheter aortic valve implantation versus surgical aortic valve replacement. *The American journal of cardiology*. Mar 15 2015;115(6):802-9. doi:10.1016/j.amjcard.2014.12.043
58. Ely EW, Girard TD, Shintani AK, et al. Apolipoprotein E4 polymorphism as a genetic predisposition to delirium in critically ill patients. *Critical care medicine*. Jan 2007;35(1):112-7. doi:10.1097/01.Ccm.0000251925.18961.Ca
59. Fan H, Ji M, Huang J, et al. Development and validation of a dynamic delirium prediction rule in patients admitted to the Intensive Care Units (DYNAMIC-ICU): A prospective cohort study. Article. *International journal of nursing studies*. 2019;93:64-73. doi:10.1016/j.ijnurstu.2018.10.008
60. Fann JR, Roth-Roemer S, Burington BE, Katon WJ, Syrjala KL. Delirium in patients undergoing hematopoietic stem cell transplantation. *Cancer*. Nov 1 2002;95(9):1971-81. doi:10.1002/cncr.10889
61. Fann JR, Hubbard RA, Alfano CM, Roth-Roemer S, Katon WJ, Syrjala KL. Pre- and post-transplantation risk factors for delirium onset and severity in patients undergoing hematopoietic stem-cell transplantation. Article. *Journal of Clinical Oncology*. 2011;29(7):895-901. doi:10.1200/JCO.2010.28.4521
62. Feast AR, White N, Lord K, Kupeli N, Vickerstaff V, Sampson EL. Pain and delirium in people with dementia in the acute general hospital setting. Article. *Age and ageing*. 2018;47(6):841-846. doi:10.1093/ageing/afy112
63. Feng Q, Ai ML, Huang L, Peng QY, Ai YH, Zhang LA. Relationship Between Cerebral Hemodynamics, Tissue Oxygen Saturation, and Delirium in Patients With Septic Shock: A Pilot Observational Cohort Study. *FRONTIERS IN MEDICINE*. 2021/11/26/ 2021;8doi:10.3389/fmed.2021.641104
64. Fick DM, Steis MR, Waller JL, Inouye SK. Delirium superimposed on dementia is associated with prolonged length of stay and poor outcomes in hospitalized older adults. *Journal of hospital medicine*. Sep 2013;8(9):500-5. doi:10.1002/jhm.2077
65. Fisher BW, Flowerdew G. A simple model for predicting postoperative delirium in older patients undergoing elective orthopedic surgery. *Journal of the American Geriatrics Society*. Feb 1995;43(2):175-8. doi:10.1111/j.1532-5415.1995.tb06385.x
66. Flink BJ, Rivelli SK, Cox EA, et al. Obstructive Sleep Apnea and Incidence of Postoperative Delirium after Elective Knee Replacement in the Nondemented Elderly. *Anesthesiology*. Apr 2012;116(4):788-796. doi:10.1097/ALN.0b013e31824b94fc
67. Fong TG, Hshieh TT, Wong B, et al. Neuropsychological Profiles of an Elderly Cohort Undergoing Elective Surgery and the Relationship Between Cognitive Performance and Delirium. *Journal of the American Geriatrics Society*. May 2015;63(5):977-982. doi:10.1111/jgs.13383

© 2023 Ormseth CH et al. *JAMA Network Open.*

68. Fortini A, Morettini A, Tavernese G, Facchini S, Tofani L, Pazzi M. Delirium in elderly patients hospitalized in internal medicine wards. *Internal and emergency medicine*. Jun 2014;9(4):435-41. doi:10.1007/s11739-013-0968-0
69. Foy A, O'Connell D, Henry D, Kelly J, Cocking S, Halliday J. Benzodiazepine use as a cause of cognitive impairment in elderly hospital inpatients. *The journals of gerontology Series A, Biological sciences and medical sciences*. Mar 1995;50(2):M99-106. doi:10.1093/gerona/50a.2.m99
70. Franco JG, Valencia C, Bernal C, et al. Relationship between cognitive status at admission and incident delirium in older medical inpatients. *The Journal of neuropsychiatry and clinical neurosciences*. Summer 2010;22(3):329-37. doi:10.1176/appi.neuropsych.22.3.329 10.1176/jnp.2010.22.3.329
71. Freter SH, George J, Dunbar MJ, Morrison M, MacKnight C, Rockwood K. Prediction of delirium in fractured neck of femur as part of routine preoperative nursing care [1]. Letter. *Age and ageing*. 2005;34(4):387-388. doi:10.1093/ageing/afi099
72. Freter S, Dunbar M, Koller K, MacKnight C, Rockwood K. Risk of Pre- and Post-Operative Delirium and the Delirium Elderly At Risk (DEAR) Tool in Hip Fracture Patients. *Canadian Geriatrics Journal*. Dec 2015;18(4):212-216. doi:10.5770/cgj.18.185
73. Galanakis P, Bickel H, Gradinger R, Von Gumppenberg S, Forstl H. Acute confusional state in the elderly following hip surgery: incidence, risk factors and complications. *International journal of geriatric psychiatry*. Apr 2001;16(4):349-55.
74. Girard TD, Ware LB, Bernard GR, et al. Associations of markers of inflammation and coagulation with delirium during critical illness. *Intensive care medicine*. Dec 2012;38(12):1965-73. doi:10.1007/s00134-012-2678-x
75. Girard TD, Thompson JL, Pandharipande PP, et al. Clinical phenotypes of delirium during critical illness and severity of subsequent long-term cognitive impairment: a prospective cohort study. *The Lancet Respiratory Medicine*. 2018/03/01/ 2018;6(3):213-222. doi:https://doi.org/10.1016/S2213-2600(18)30062-6
76. Giroux M, Sirois MJ, Boucher V, et al. Frailty Assessment to Help Predict Patients at Risk of Delirium When Consulting the Emergency Department. *The Journal of emergency medicine*. Aug 2018;55(2):157-164. doi:10.1016/j.jemermed.2018.02.032
77. GOLDENBERG G, KISELEV P, BHARATHAN T, et al. Predicting post-operative delirium in elderly patients undergoing surgery for hip fracture. *Psychogeriatrics : the official journal of the Japanese Psychogeriatric Society*. 2006;6(2):43-48.
78. Goudzwaard JA, de Ronde-Tillmans M, de Jager TAJ, et al. Incidence, determinants and consequences of delirium in older patients after transcatheter aortic valve implantation. *AGE AND AGEING*. 2020/05//undefined 2020;49(3):389-394. doi:10.1093/ageing/afaa001
79. Greene NH, Attix DK, Weldon BC, Smith PJ, McDonagh DL, Monk TG. Measures of executive function and depression identify patients at risk for postoperative delirium. *Anesthesiology*. Apr 2009;110(4):788-95. doi:10.1097/aln.0b013e31819b5ba6
80. WJ G, JX Z, RQ J, LY Z, CM W. - Incidence, risk factors, and consequences of emergence delirium after elective. *Surgeon*. 2022;20(5):e214-e220.
81. Gual N, Morandi A, Perez LM, et al. Risk Factors and Outcomes of Delirium in Older Patients Admitted to Postacute Care with and without Dementia. *Dementia and geriatric cognitive disorders*. 2018;45(1-2):121-129. doi:10.1159/000485794

© 2023 Ormseth CH et al. *JAMA Network Open.*

82. Guo Y, Jia P, Zhang J, Wang X, Jiang H, Jiang W. Prevalence and risk factors of postoperative delirium in elderly hip fracture patients. *The Journal of international medical research*. Apr 2016;44(2):317-27. doi:10.1177/0300060515624936
83. Guo Z, Liu J, Li J, et al. Postoperative Delirium in Severely Burned Patients Undergoing Early Escharotomy: Incidence, Risk Factors, and Outcomes. *Journal of burn care & research : official publication of the American Burn Association*. Jan/Feb 2017;38(1):e370-e376. doi:10.1097/bcr.0000000000000397
84. Hall RJ, Watne LO, Idland AV, et al. Cerebrospinal fluid levels of neopterin are elevated in delirium after hip fracture. *Journal of Neuroinflammation*. Jun 2016;13170. doi:10.1186/s12974-016-0636-1
85. Hatta K, Kishi Y, Wada K, et al. Real-World Effectiveness of Ramelteon and Suvorexant for Delirium Prevention in 948 Patients With Delirium Risk Factors. *The Journal of clinical psychiatry*. 2019/12/17/ 2019;81(1):19m12865. doi:10.4088/JCP.19m12865
86. Hayhurst CJ, Patel MB, McNeil JB, et al. Association of neuronal repair biomarkers with delirium among survivors of critical illness. *JOURNAL OF CRITICAL CARE*. 2020/04//undefined 2020;56:94-99. doi:10.1016/j.jcrc.2019.12.010
87. He R, Wang F, Shen HR, Zeng Y, Zhang LJ. Association between increased neutrophil-to-lymphocyte ratio and postoperative delirium in elderly patients with total hip arthroplasty for hip fracture. *BMC PSYCHIATRY*. 2020/10/07/ 2020;20(1)doi:10.1186/s12888-020-02908-2
88. Hein C, Forgues A, Piau A, Sommet A, Nourhashémi F, Vellas B. Impact of Polypharmacy on Occurrence of Delirium in Elderly Emergency Patients. Article. *Journal of the American Medical Directors Association*. 2014;15(11):850e11-850e15. doi:10.1016/j.jamda.2014.08.012
89. Heng M, Eagen CE, Javedan H, Kodela J, Weaver MJ, Harris MB. Abnormal Mini-Cog Is Associated with Higher Risk of Complications and Delirium in Geriatric Patients with Fracture. *The Journal of bone and joint surgery American volume*. May 4 2016;98(9):742-50. doi:10.2106/jbjs.15.00859
90. Henjum K, Quist-Paulsen E, Zetterberg H, Blennow K, Nilsson LNG, Watne LO. CSF sTREM2 in deliriumrelation to Alzheimer's disease CSF biomarkers A beta 42, t-tau and p-tau. *Journal of Neuroinflammation*. Nov 2018;15304. doi:10.1186/s12974-018-1331-1
91. Hirsch J, DePalma G, Tsai TT, Sands LP, Leung JM. Impact of intraoperative hypotension and blood pressure fluctuations on early postoperative delirium after non-cardiac surgery. *British journal of anaesthesia*. Sep 2015;115(3):418-26. doi:10.1093/bja/aeu458
92. Hsieh SJ, Soto GJ, Hope AA, Ponea A, Gong MN. The association between acute respiratory distress syndrome, delirium, and in-hospital mortality in intensive care unit patients. *American journal of respiratory and critical care medicine*. Jan 1 2015;191(1):71-8. doi:10.1164/rccm.201409-1690OC
93. Huang HW, Zhang GB, Li HY, et al. Development of an early prediction model for postoperative delirium in neurosurgical patients admitted to the ICU after elective craniotomy (E-PREPOD-NS): A secondary analysis of a prospective cohort study. *JOURNAL OF CLINICAL NEUROSCIENCE*. 2021/08//undefined 2021;90:217-224. doi:10.1016/j.jocn.2021.06.004
94. Hughes CG, Morandi A, Girard TD, et al. Association between Endothelial Dysfunction and Acute Brain Dysfunction during Critical Illness. *Anesthesiology*. Mar 2013;118(3):631-639. doi:10.1097/ALN.0b013e31827bd193

© 2023 Ormseth CH et al. *JAMA Network Open.*

95. Hughes CG, Pandharipande PP, Thompson JL, et al. Endothelial Activation and Blood-Brain Barrier Injury as Risk Factors for Delirium in Critically Ill Patients. *Critical care medicine*. Sep 2016;44(9):e809-17. doi:10.1097/ccm.0000000000001739
96. Humbert M, Büla CJ, Muller O, Krief H, Monney P. Delirium in older patients undergoing aortic valve replacement: incidence, predictors, and cognitive prognosis. *BMC geriatrics*. 2021/03/02/ 2021;21(1):153. doi:10.1186/s12877-021-02100-5
97. Hwang H, Lee KM, Son KL, et al. Incidence and risk factors of subsyndromal delirium after curative resection of gastric cancer. *BMC cancer*. Jul 27 2018;18(1):765. doi:10.1186/s12885-018-4681-2
98. Iamaroon A, Wongviriyawong T, Sura-arunsumrit P, Wiwatnodom N, Rewuri N, Chaiwat O. Incidence of and risk factors for postoperative delirium in older adult patients undergoing noncardiac surgery: a prospective study. *BMC GERIATRICS*. 2020/02/03/ 2020;20(1)doi:10.1186/s12877-020-1449-8
99. Idland AV, Wyller TB, Stoen R, et al. Preclinical Amyloid-beta and Axonal Degeneration Pathology in Delirium. *Journal of Alzheimer's disease : JAD*. 2017;55(1):371-379. doi:10.3233/jad-160461
100. Inouye SK, Viscoli CM, Horwitz RI, Hurst LD, Tinetti ME. A predictive model for delirium in hospitalized elderly medical patients based on admission characteristics. *Annals of internal medicine*. Sep 15 1993;119(6):474-81. doi:10.7326/0003-4819-119-6-199309150-00005
101. Inouye SK, Charpentier PA. Precipitating factors for delirium in hospitalized elderly persons - Predictive model and interrelationship with baseline vulnerability. *Jama-Journal of the American Medical Association*. Mar 1996;275(11):852-857. doi:10.1001/jama.275.11.852
102. Inouye SK, Zhang Y, Jones RN, Kiely DK, Yang F, Marcantonio ER. Risk factors for delirium at discharge - Development and validation of a predictive model. *Archives of internal medicine*. Jul 2007;167(13):1406-1413. doi:10.1001/archinte.167.13.1406
103. Jankowski CJ, Trenerry MR, Cook DJ, et al. Cognitive and functional predictors and sequelae of postoperative delirium in elderly patients undergoing elective joint arthroplasty. *Anesthesia and analgesia*. May 2011;112(5):1186-93. doi:10.1213/ANE.0b013e318211501b
104. Jones RN, Yang FM, Zhang Y, Kiely DK, Marcantonio ER, Inouye SK. Does Educational Attainment Contribute to Risk for Delirium? A Potential Role for Cognitive Reserve. *The Journals of Gerontology: Series A: Biological Sciences and Medical Sciences*. Dec 2006 2017-09-25 2006;61(12):1307-1311. doi:http://dx.doi.org/10.1093/gerona/61.12.1307
105. Joosten E, Lemiengre J, Nelis T, Verbeke G, Milisen K. Is anaemia a risk factor for delirium in an acute geriatric population? *Gerontology*. 2006;52(6):382-5. doi:10.1159/000095126
106. Joosten E, Demuynck M, Detroyer E, Milisen K. Prevalence of frailty and its ability to predict in hospital delirium, falls, and 6-month mortality in hospitalized older patients. *BMC geriatrics*. Jan 6 2014;14:1. doi:10.1186/1471-2318-14-1
107. Juliebo V, Bjoro K, Krogseth M, Skovlund E, Ranhoff AH, Wyller TB. Risk factors for preoperative and postoperative delirium in elderly patients with hip fracture. *Journal of the American Geriatrics Society*. Aug 2009;57(8):1354-61. doi:10.1111/j.1532-5415.2009.02377.x
108. Kagansky N, Rimon E, Naor S, Dvornikov E, Cojocaru L, Levy S. Low incidence of delirium in very old patients after surgery for hip fractures. *The American Journal of Geriatric Psychiatry*. Jun 2004

© 2023 Ormseth CH et al. *JAMA Network Open.*

2018-11-15 2004;12(3):306-314. doi:http://dx.doi.org/10.1176/appi.ajgp.12.3.306
109. Kalisvaart KJ, Vreeswijk R, de Jonghe JF, van der Ploeg T, van Gool WA, Eikelenboom P. Risk factors and prediction of postoperative delirium in elderly hip-surgery patients: implementation and validation of a medical risk factor model. *Journal of the American Geriatrics Society*. May 2006;54(5):817-22. doi:10.1111/j.1532-5415.2006.00704.x
110. Kang T, Park SY, Lee JH, et al. Incidence & Risk Factors of Postoperative Delirium After Spinal Surgery in Older Patients. *Scientific reports*. 2020/06/08/ 2020;10(1):9232. doi:10.1038/s41598-020-66276-3
111. Kanova M, Sklienka P, Roman K, Burda M, Janoutova J. Incidence and risk factors for delirium development in ICU patients - a prospective observational study. *Biomedical papers of the Medical Faculty of the University Palacky, Olomouc, Czechoslovakia*. Jun 2017;161(2):187-196. doi:10.5507/bp.2017.004
112. Kazmierski J, Kowman M, Banach M, et al. Incidence and predictors of delirium after cardiac surgery: Results from The IPDACS Study. *Journal of psychosomatic research*. Aug 2010;69(2):179-85. doi:10.1016/j.jpsychores.2010.02.009
113. Kazmierski J, Banys A, Latek J, Bourke J, Jaszewski R. Cortisol levels and neuropsychiatric diagnosis as markers of postoperative delirium: a prospective cohort study. *Critical care (London, England)*. Mar 1 2013;17(2):R38. doi:10.1186/cc12548
114. Kazmierski J, Banys A, Latek J, Bourke J, Jaszewski R. Raised IL-2 and TNF-alpha concentrations are associated with postoperative delirium in patients undergoing coronary-artery bypass graft surgery. *International psychogeriatrics*. May 2014;26(5):845-55. doi:10.1017/s1041610213002378
115. Kazmierski J, Banys A, Latek J, et al. Mild cognitive impairment with associated inflammatory and cortisol alterations as independent risk factor for postoperative delirium. Article. *Dementia and geriatric cognitive disorders*. 2014;38(1-2):65-78. doi:10.1159/000357454
116. Kazmierski J, Sieruta M, Banys A, et al. The assessment of the T102C polymorphism of the 5HT2a receptor gene, 3723G/A polymorphism of the NMDA receptor 3A subunit gene (GRIN3A) and 421C/A polymorphism of the NMDA receptor 2B subunit gene (GRIN2B) among cardiac surgery patients with and without delirium. *General hospital psychiatry*. Nov-Dec 2014;36(6):753-756. doi:10.1016/j.genhosppsych.2014.06.002
117. Kennedy M, Enander RA, Tadiri SP, Wolfe RE, Shapiro NI, Marcantonio ER. Delirium risk prediction, healthcare use and mortality of elderly adults in the emergency department. *Journal of the American Geriatrics Society*. Mar 2014;62(3):462-9. doi:10.1111/jgs.12692
118. Khan BA, Perkins A, Hui SL, et al. Relationship Between African-American Race and Delirium in the ICU. *Critical care medicine*. Sep 2016;44(9):1727-34. doi:10.1097/ccm.0000000000001813
119. Khan BA, Perkins AJ, Prasad NK, et al. Biomarkers of Delirium Duration and Delirium Severity in the ICU. *Critical care medicine*. 2020/03//undefined 2020;48(3):353-361. doi:10.1097/CCM.0000000000004139
120. Khan SH, Lindroth H, Hendrie K, et al. Time trends of delirium rates in the intensive care unit. *HEART & LUNG*. 2020/09//undefined 2020;49(5):572-577. doi:10.1016/j.hrtlng.2020.03.006

© 2023 Ormseth CH et al. *JAMA Network Open.*

121. Kim MY, Park UJ, Kim HT, Cho WH. DELirium Prediction Based on Hospital Information (Delphi) in General Surgery Patients. *Medicine*. Mar 2016;95(12):e3072. doi:10.1097/md.0000000000003072
122. Kim JY, Yoo JH, Kim E, et al. Risk factors and clinical outcomes of delirium in osteoporotic hip fractures. *Journal of orthopaedic surgery (Hong Kong)*. Sep-Dec 2017;25(3):2309499017739485. doi:10.1177/2309499017739485
123. Kim KH, Kang SY, Shin DA, et al. Parkinson's disease-related non-motor features as risk factors for post-operative delirium in spinal surgery. *PloS one*. 2018;13(4):e0195749. doi:10.1371/journal.pone.0195749
124. Kim Y, Jin Y, Jin T, Lee S-M. Risk factors and outcomes of sepsis-associated delirium in intensive care unit patients: A secondary data analysis. *Intensive & critical care nursing*. 2020/08//undefined 2020;59:102844. doi:10.1016/j.iccn.2020.102844
125. Kong S, Wang J, Xu H, Wang K. Effect of hypertension and medication use regularity on postoperative delirium after maxillofacial tumors radical surgery. *Oncotarget*. Aug 31 2021;12(18):1811-1820. doi:10.18632/oncotarget.28048
126. Korevaar JC, van Munster BC, de Rooij SE. Risk factors for delirium in acutely admitted elderly patients: a prospective cohort study. *BMC geriatrics*. Apr 13 2005;5:6. doi:10.1186/1471-2318-5-6
127. Kosar CM, Tabloski PA, Travison TG, et al. Effect of preoperative pain and depressive symptoms on the risk of postoperative delirium: a prospective cohort study. *Lancet Psychiatry*. Nov 2014;1(6):431-436. doi:10.1016/s2215-0366(14)00006-6
128. Koskderelioglu A, Onder O, Gucuyener M, Altay T, Kayali C, Gedizlioglu M. Screening for postoperative delirium in patients with acute hip fracture: Assessment of predictive factors. *Geriatrics & gerontology international*. Jun 2017;17(6):919-924. doi:10.1111/ggi.12806
129. Kostalova M, Bednarik J, Mitasova A, et al. Towards a predictive model for post-stroke delirium. *Brain injury*. 2012;26(7-8):962-71. doi:10.3109/02699052.2012.660510
130. Krzych LJ, Wybraniec MT, Krupka-Matuszczyk I, et al. Complex assessment of the incidence and risk factors of delirium in a large cohort of cardiac surgery patients: a single-center 6-year experience. *BioMed research international*. 2013;2013:835850. doi:10.1155/2013/835850
131. Kumar AK, Jayant A, Arya VK, Magoon R, Sharma R. Delirium after cardiac surgery: A pilot study from a single tertiary referral center. *Annals of cardiac anaesthesia*. Jan-Mar 2017;20(1):76-82. doi:10.4103/0971-9784.197841
132. Kupiec A, Adamik B, Kozera N, Gozdzik W. Elevated Procalcitonin as a Risk Factor for Postoperative Delirium in the Elderly after Cardiac Surgery-A Prospective Observational Study. *JOURNAL OF CLINICAL MEDICINE*. 2020/12//undefined 2020;9(12)doi:10.3390/jcm9123837
133. Kwizera A, Nakibuuka J, Ssemogerere L, et al. Incidence and Risk Factors for Delirium among Mechanically Ventilated Patients in an African Intensive Care Setting: An Observational Multicenter Study. *Critical Care Research and Practice*. 2015;Unsp 491780. doi:10.1155/2015/491780
134. Lai MM, Wong Tin Niam DM. Intracranial cause of delirium: computed tomography yield and predictive factors. *Internal medicine journal*. Apr 2012;42(4):422-7. doi:10.1111/j.1445-5994.2010.02400.x

© 2023 Ormseth CH et al. *JAMA Network Open.*

135. Lee HB, Mears SC, Rosenberg PB, Leoutsakos JM, Gottschalk A, Sieber FE. Predisposing factors for postoperative delirium after hip fracture repair in individuals with and without dementia. *Journal of the American Geriatrics Society*. Dec 2011;59(12):2306-13. doi:10.1111/j.1532-5415.2011.03725.x
136. Lee KH, Ha YC, Lee YK, Kang H, Koo KH. Frequency, risk factors, and prognosis of prolonged delirium in elderly patients after hip fracture surgery. *Clinical orthopaedics and related research*. Sep 2011;469(9):2612-20. doi:10.1007/s11999-011-1806-1
137. Lee SS, Lo Y, Verghese J. Physical Activity and Risk of Postoperative Delirium. *Journal of the American Geriatrics Society*. 2019/11//undefined 2019;67(11):2260-2266. doi:10.1111/jgs.16083
138. Leung JM, Sands LP, Mullen EA, Wang Y, Vaurio L. Are Preoperative Depressive Symptoms Associated With Postoperative Delirium in Geriatric Surgical Patients? *The Journals of Gerontology: Series A*. 2005;60(12):1563-1568. doi:10.1093/gerona/60.12.1563
139. Leung JM, Sands LP, Wang Y, et al. Apolipoprotein E e4 allele increases the risk of early postoperative delirium in older patients undergoing noncardiac surgery. *Anesthesiology*. Sep 2007;107(3):406-11. doi:10.1097/01.anes.0000278905.07899.df
140. Leung JM, Tsai TL, Sands LP. Brief report: preoperative frailty in older surgical patients is associated with early postoperative delirium. *Anesthesia and analgesia*. May 2011;112(5):1199-201. doi:10.1213/ANE.0b013e31820c7c06
141. Leung JM, Sands LP, Lim E, Tsai TL, Kinjo S. Does preoperative risk for delirium moderate the effects of postoperative pain and opiate use on postoperative delirium? *The American journal of geriatric psychiatry : official journal of the American Association for Geriatric Psychiatry*. Oct 2013;21(10):946-56. doi:10.1016/j.jagp.2013.01.069
142. Levkoff SE, Evans DA, Liptzin B, et al. Delirium. The occurrence and persistence of symptoms among elderly hospitalized patients. *Archives of internal medicine*. Feb 1992;152(2):334-40.
143. Lewis EG, Banks J, Paddick SM, et al. Risk Factors for Delirium in Older Medical Inpatients in Tanzania. Article. *Dementia and geriatric cognitive disorders*. 2017;44(3-4):160-170. doi:10.1159/000479058
144. Li G, Lei X, Ai C, Li T, Chen Z. Low plasma leptin level at admission predicts delirium in critically ill patients: A prospective cohort study. *Peptides*. Jul 2017;93:27-32. doi:10.1016/j.peptides.2017.05.002
145. Li X, Zhang L, Gong F, Ai Y. Incidence and Risk Factors for Delirium in Older Patients Following Intensive Care Unit Admission: A Prospective Observational Study. *The journal of nursing research : JNR*. 2020/08//undefined 2020;28(4):e101. doi:10.1097/jnr.0000000000000384
146. Liang CK, Chu CL, Chou MY, et al. Developing a Prediction Model for Post-Operative Delirium and Long-Term Outcomes Among Older Patients Receiving Elective Orthopedic Surgery: A Prospective Cohort Study in Taiwan. *Rejuvenation Research*. Aug 2015;18(4):347-355. doi:10.1089/rej.2014.1645
147. Lin Y, Chen Q, Zhang H, et al. Risk factors for postoperative delirium in patients with triple-branched stent graft implantation. *Journal of cardiothoracic surgery*. 2020/07/14/ 2020;15(1):171. doi:10.1186/s13019-020-01217-9

© 2023 Ormseth CH et al. *JAMA Network Open.*

148. Lin X, Tang J, Liu C, et al. Cerebrospinal fluid cholinergic biomarkers are associated with postoperative delirium in elderly patients undergoing Total hip/knee replacement: a prospective cohort study. *BMC anesthesiology*. 2020/09/28/ 2020;20(1):246. doi:10.1186/s12871-020-01166-9
149. Lin X, Liu F, Wang B, et al. Subjective cognitive decline may be associated with post-operative delirium in patients undergoing total hip replacement: The PNDABLE study. *Frontiers in Aging Neuroscience*. 2021/06/11/
2021-07-29</p> 2021;13:8. doi:10.3389/fnagi.2021.680672
150. Limpawattana P, Panitchote A, Tangvoraphonkchai K, et al. Delirium in critical care: a study of incidence, prevalence, and associated factors in the tertiary care hospital of older Thai adults. *Aging & Mental Health*. 2016/01/02 2016;20(1):74-80. doi:10.1080/13607863.2015.1035695
151. Lindroth H, Bratzke L, Twadell S, et al. Predicting postoperative delirium severity in older adults: The role of surgical risk and executive function. *International journal of geriatric psychiatry*. Jul 2019;34(7):1018-1028. doi:10.1002/gps.5104
152. Litaker D, Locala J, Franco K, Bronson DL, Tannous Z. Preoperative risk factors for postoperative delirium. *General hospital psychiatry*. Mar-Apr 2001;23(2):84-9.
153. Ma JR, Fan MM, Wang ZS. Age, preoperative higher serum cortisol levels, and lower serum acetylcholine levels predict delirium after percutaneous coronary intervention in acute coronary syndrome patients accompanied with renal dysfunction. *INDIAN JOURNAL OF PSYCHIATRY*. 2020/03//undefined 2020;62(2):172-177. doi:10.4103/psychiatry.IndianJPsychiatry_37_19
154. Ma J, Li C, Zhang W, et al. Preoperative anxiety predicted the incidence of postoperative delirium in patients undergoing total hip arthroplasty: a prospective cohort study. *BMC anesthesiology*. 2021/02/12/ 2021;21(1):48. doi:10.1186/s12871-021-01271-3
155. Mahanna-Gabrielli E, Zhang K, Sieber FE, et al. Frailty Is Associated With Postoperative Delirium But Not With Postoperative Cognitive Decline in Older Noncardiac Surgery Patients. *ANESTHESIA AND ANALGESIA*. 2020/06//undefined 2020;130(6):1516-1523. doi:10.1213/ANE.0000000000004773
156. Marcantonio ER, Juarez G, Goldman L, et al. The relationship of postoperative delirium with psychoactive medications. *Jama*. Nov 16 1994;272(19):1518-22.
157. Marcantonio ER, Goldman L, Orav EJ, Cook EF, Lee TH. The association of intraoperative factors with the development of postoperative delirium. *The American journal of medicine*. Nov 1998;105(5):380-4. doi:10.1016/s0002-9343(98)00292-7
158. Martin NJ, Stones MJ, Young JE, Bedard M. Development of delirium: a prospective cohort study in a community hospital. *International psychogeriatrics*. Mar 2000;12(1):117-27.
159. Martinez JA, Belastegui A, Basabe I, et al. Derivation and validation of a clinical prediction rule for delirium in patients admitted to a medical ward: an observational study. *BMJ open*. 2012;2(5)e001599. doi:10.1136/bmjopen-2012-001599
160. Matsuda Y, Maeda I, Morita T, et al. Reversibility of delirium in Ill-hospitalized cancer patients: Does underlying etiology matter? *Cancer medicine*. 2020/01//undefined 2020;9(1):19-26. doi:10.1002/cam4.2669

© 2023 Ormseth CH et al. *JAMA Network Open.*

161. Matsuo N, Morita T, Matsuda Y, et al. Predictors of Delirium in Corticosteroid-Treated Patients with Advanced Cancer: An Exploratory, Multicenter, Prospective, Observational Study. *Journal of palliative medicine*. Apr 2017;20(4):352-359. doi:10.1089/jpm.2016.0323
162. Mazzola P, Ward L, Zazzetta S, et al. Association Between Preoperative Malnutrition and Postoperative Delirium After Hip Fracture Surgery in Older Adults. *Journal of the American Geriatrics Society*. Jun 2017;65(6):1222-1228. doi:10.1111/jgs.14764
163. McAlpine JN, Hodgson EJ, Abramowitz S, et al. The incidence and risk factors associated with postoperative delirium in geriatric patients undergoing surgery for suspected gynecologic malignancies. *Gynecologic oncology*. May 2008;109(2):296-302. doi:10.1016/j.ygyno.2008.02.016
164. McAvay GJ, Van Ness PH, Bogardus ST, Jr., et al. Depressive symptoms and the risk of incident delirium in older hospitalized adults. *Journal of the American Geriatrics Society*. May 2007;55(5):684-91. doi:10.1111/j.1532-5415.2007.01150.x
165. McCusker J, Cole MG, Voyer P, et al. Prevalence and incidence of delirium in long-term care. *International journal of geriatric psychiatry*. Nov 2011;26(11):1152-61. doi:10.1002/gps.2654
166. McManus J, Pathansali R, Hassan H, et al. The course of delirium in acute stroke. *Age and ageing*. Jul 2009;38(4):385-9. doi:10.1093/ageing/afp038
167. McNicoll L, Pisani MA, Zhang Y, Ely EW, Siegel MD, Inouye SK. Delirium in the intensive care unit: Occurrence and clinical course in older patients. *Journal of the American Geriatrics Society*. May 2003;51(5):591-598. doi:10.1034/j.1600-0579.2003.00201.x
168. McPherson JA, Wagner CE, Boehm LM, et al. Delirium in the cardiovascular ICU: exploring modifiable risk factors. *Critical care medicine*. Feb 2013;41(2):405-13. doi:10.1097/CCM.0b013e31826ab49b
169. Meziere A, Paillaud E, Belmin J, et al. Delirium in older people after proximal femoral fracture repair: role of a preoperative screening cognitive test. *Annales francaises d'anesthesie et de reanimation*. Sep 2013;32(9):e91-6. doi:10.1016/j.annfar.2013.06.006
170. Miao S, Shen P, Zhang Q, et al. Neopterin and mini-mental state examination scores, two independent risk factors for postoperative delirium in elderly patients with open abdominal surgery. *Journal of cancer research and therapeutics*. Oct-Dec 2018;14(6):1234-1238. doi:10.4103/0973-1482.192764
171. Miu DK, Yeung JC. Incidence of post-stroke delirium and 1-year outcome. *Geriatrics & gerontology international*. Jan 2013;13(1):123-9. doi:10.1111/j.1447-0594.2012.00871.x
172. Miu DK, Chan CW, Kok C. Delirium among elderly patients admitted to a post-acute care facility and 3-months outcome. *Geriatrics & gerontology international*. May 2016;16(5):586-92. doi:10.1111/ggi.12521
173. Moorey HC, Zaidman S, Jackson TA. Delirium is not associated with anticholinergic burden or polypharmacy in older patients on admission to an acute hospital: an observational case control study. *BMC geriatrics*. Sep 2016;16162. doi:10.1186/s12877-016-0336-9
174. Morandi A, Gunther ML, Pandharipande PP, et al. Insulin-like growth factor-1 and delirium in critically ill mechanically ventilated patients: a preliminary investigation. *International psychogeriatrics*. Sep 2011;23(7):1175-81. doi:10.1017/s1041610210002486

© 2023 Ormseth CH et al. *JAMA Network Open.*

175. Morandi A, Barnett N, Miller RR, 3rd, et al. Vitamin D and delirium in critically ill patients: a preliminary investigation. *Journal of critical care*. Jun 2013;28(3):230-5. doi:10.1016/j.jcrc.2012.06.011
176. Morandi A, Hughes CG, Thompson JL, et al. Statins and delirium during critical illness: a multicenter, prospective cohort study. *Critical care medicine*. Aug 2014;42(8):1899-909. doi:10.1097/ccm.0000000000000398
177. Mori S, Takeda JRT, Carrara FSA, Cohrs CR, Zanei SSV, Whitaker IY. Incidence and factors related to delirium in an Intensive Care Unit. *Revista Da Escola De Enfermagem Da Usp*. Jul-Aug 2016;50(4):585-591. doi:10.1590/s0080-623420160000500014
178. Morrison RS, Magaziner J, Gilbert M, et al. Relationship between pain and opioid analgesics on the development of delirium following hip fracture. Article. *Journals of Gerontology - Series A Biological Sciences and Medical Sciences*. 2003;58(1):76-81.
179. Mu DL, Wang DX, Li LH, et al. High serum cortisol level is associated with increased risk of delirium after coronary artery bypass graft surgery: a prospective cohort study. *Critical care (London, England)*. 2010;14(6):R238. doi:10.1186/cc9393
180. Muangpaisan W, Wongprikron A, Srinonprasert V, Suwanpatoomlerd S, Sutipornpalangkul W, Assantchai P. Incidence and risk factors of acute delirium in older patients with hip fracture in Siriraj Hospital. *Journal of the Medical Association of Thailand = Chotmaihet thangphaet*. Apr 2015;98(4):423-30.
181. Neerland BE, Krogseth M, Juliebo V, et al. Perioperative hemodynamics and risk for delirium and new onset dementia in hip fracture patients; A prospective follow-up study. *PloS one*. 2017;12(7):e0180641. doi:10.1371/journal.pone.0180641
182. Nekrosius D, Kaminskaite M, Jokubka R, et al. Association of COMT Val158Met Polymorphism With Delirium Risk and Outcomes After Traumatic Brain Injury. Article in Press. *The Journal of neuropsychiatry and clinical neurosciences*. 2019:appineuropsych18080195. doi:10.1176/appi.neuropsych.18080195
183. Neufeld KJ, Leoutsakos JS, Sieber FE, et al. Evaluation of two delirium screening tools for detecting post-operative delirium in the elderly. *British journal of anaesthesia*. Oct 2013;111(4):612-8. doi:10.1093/bja/aet167
184. Ngo LH, Inouye SK, Jones RN, et al. Methodologic considerations in the design and analysis of nested case-control studies: association between cytokines and postoperative delirium. *BMC medical research methodology*. Jun 2017;1788. doi:10.1186/s12874-017-0359-8
185. Nie H, Zhao B, Zhang YQ, Jiang YH, Yang YX. Pain and cognitive dysfunction are the risk factors of delirium in elderly hip fracture Chinese patients. *Archives of gerontology and geriatrics*. Mar-Apr 2012;54(2):e172-4. doi:10.1016/j.archger.2011.09.012
186. Noriega FJ, Vidan MT, Sanchez E, et al. Incidence and impact of delirium on clinical and functional outcomes in older patients hospitalized for acute cardiac diseases. *American heart journal*. Nov 2015;170(5):938-44. doi:10.1016/j.ahj.2015.08.007
187. Norkiene I, Ringaitiene D, Misiuriene I, et al. Incidence and precipitating factors of delirium after coronary artery bypass grafting. *Scandinavian cardiovascular journal : SCJ*. Jun 2007;41(3):180-5. doi:10.1080/14017430701302490
188. O'Regan NA, Fitzgerald J, Adamis D, Molloy DW, Meagher D, Timmons S. Predictors of delirium development in older medical inpatients: Readily identifiable factors at admission. Article. *Journal of Alzheimer's Disease*. 2018;64(3):775-785. doi:10.3233/JAD-180178

© 2023 Ormseth CH et al. *JAMA Network Open.*

189. Oh ES, Sieber FE, Leoutsakos JM, Inouye SK, Lee HB. Sex Differences in Hip Fracture Surgery: Preoperative Risk Factors for Delirium and Postoperative Outcomes. *Journal of the American Geriatrics Society*. Aug 2016;64(8):1616-21. doi:10.1111/jgs.14243
190. Ojagbemi A, Owolabi M, Bello T, Baiyewu O. Stroke severity predicts poststroke delirium and its association with dementia: Longitudinal observation from a low income setting. *Journal of the neurological sciences*. Apr 15 2017;375:376-381. doi:10.1016/j.jns.2017.02.039
191. Oldham MA, Hawkins KA, Lin IH, et al. Depression Predicts Delirium After Coronary Artery Bypass Graft Surgery Independent of Cognitive Impairment and Cerebrovascular Disease: An Analysis of the Neuropsychiatric Outcomes After Heart Surgery Study. Article. *American Journal of Geriatric Psychiatry*. 2019;27(5):476-486. doi:10.1016/j.jagp.2018.12.025
192. Oliveira FR, Oliveira VH, Oliveira M, et al. Hypertension, mitral valve disease, atrial fibrillation and low education level predict delirium and worst outcome after cardiac surgery in older adults. Article. *BMC anesthesiology*. 2018;18(1)doi:10.1186/s12871-018-0481-0
193. Osse RJ, Fekkes D, Tulen JHM, et al. High Preoperative Plasma Neopterin Predicts Delirium After Cardiac Surgery in Older Adults. *Journal of the American Geriatrics Society*. Apr 2012;60(4):661-668. doi:10.1111/j.1532-5415.2011.03885.x
194. Otomo S, Maekawa K, Goto T, Baba T, Yoshitake A. Pre-existing cerebral infarcts as a risk factor for delirium after coronary artery bypass graft surgery. *Interactive cardiovascular and thoracic surgery*. Nov 2013;17(5):799-804. doi:10.1093/icvts/ivt304
195. Pandharipande P, Shintani A, Peterson J, et al. Lorazepam is an independent risk factor for transitioning to delirium in intensive care unit patients. *Anesthesiology*. Jan 2006;104(1):21-6. doi:10.1097/00000542-200601000-00005
196. Pandharipande PP, Morandi A, Adams JR, et al. Plasma tryptophan and tyrosine levels are independent risk factors for delirium in critically ill patients. *Intensive care medicine*. Nov 2009;35(11):1886-92. doi:10.1007/s00134-009-1573-6
197. Park H, Kim KW, Yoon IY. Smoking Cessation and the Risk of Hyperactive Delirium in Hospitalized Patients: A Retrospective Study. *Canadian journal of psychiatry Revue canadienne de psychiatrie*. Oct 2016;61(10):643-51. doi:10.1177/0706743716652401
198. Pasinska P, Kowalska K, Klimiec E, Szyper-Maciejowska A, Wilk A, Klimkowicz-Mrowiec A. Frequency and predictors of post-stroke delirium in PRospective Observational POLIsh Study (PROPOLIS). *Journal of neurology*. Apr 2018;265(4):863-870. doi:10.1007/s00415-018-8782-2
199. Patrono D, Rigo F, Bormida S, et al. Graft factors as determinants of postoperative delirium after liver transplantation. *Updates in surgery*. 2020/12//undefined 2020;72(4):1053-1063. doi:10.1007/s13304-020-00887-3
200. Pedemonte JC, Plummer GS, Chamadia S, et al. Electroencephalogram Burst-suppression during Cardiopulmonary Bypass in Elderly Patients Mediates Postoperative Delirium. *ANESTHESIOLOGY*. 2020/08//undefined 2020;133(2):280-292. doi:10.1097/ALN.0000000000003328
201. Pendlebury ST, Lovett NG, Smith SC, et al. Observational, longitudinal study of delirium in consecutive unselected acute medical admissions: age-specific rates and associated factors, mortality and re-admission. *BMJ open*. 2015;5(11)e007808. doi:10.1136/bmjopen-2015-007808
202. Peng J, Wu G, Chen J, Chen H. Preoperative C-Reactive Protein/Albumin Ratio, a Risk Factor for Postoperative Delirium in Elderly Patients After Total Joint Arthroplasty. *The Journal of arthroplasty*. 2019/11//undefined 2019;34(11):2601-2605. doi:10.1016/j.arth.2019.06.042

© 2023 Ormseth CH et al. *JAMA Network Open.*

203. Pérez-Ros P, Martínez-Arnau FM, Baixauli-Alacreu S, Caballero-Pérez M, García-Gollarte JF, Tarazona-Santabalbina F. Delirium Predisposing and Triggering Factors in Nursing Home Residents: A Cohort Trial-Nested Case-Control Study. *Journal of Alzheimer's disease : JAD*. 2019 2019;70(4):1113-1122. doi:10.3233/JAD-190391
204. Pioli G, Bendini C, Giusti A, et al. Surgical delay is a risk factor of delirium in hip fracture patients with mild-moderate cognitive impairment. *Aging clinical and experimental research*. Jan 2019;31(1):41-47. doi:10.1007/s40520-018-0985-y
205. Pipanmekaporn T, Punjasawadwong Y, Wongpakaran N, et al. Risk factors and adverse clinical outcomes of postoperative delirium in Thai elderly patients: A prospective cohort study. *Perspectives in psychiatric care*. 2021/07//undefined 2021;57(3):1073-1082. doi:10.1111/ppc.12658
206. Pisani MA, Araujo KL, Murphy TE. Association of cumulative dose of haloperidol with next-day delirium in older medical ICU patients. *Critical care medicine*. May 2015;43(5):996-1002. doi:10.1097/ccm.0000000000000863
207. Pollmann CT, Mellingsaeter MR, Neerland BE, Straume-Naesheim T, Aroen A, Watne LO. Orthogeriatric co-management reduces incidence of delirium in hip fracture patients. *OSTEOPOROSIS INTERNATIONAL*. 2021/11//undefined 2021;32(11):2225-2233. doi:10.1007/s00198-021-05974-8
208. Pompei P, Foreman M, Rudberg MA, Inouye SK, Braund V, Cassel CK. Delirium in hospitalized older persons: outcomes and predictors. *Journal of the American Geriatrics Society*. Aug 1994;42(8):809-15. doi:10.1111/j.1532-5415.1994.tb06551.x
209. Qi J, Liu C, Chen La, Chen J. Postoperative Serum Albumin Decrease Independently Predicts Delirium in the Elderly Subjects after Total Joint Arthroplasty. *Current pharmaceutical design*. 2020 2020;26(3):386-394. doi:10.2174/1381612826666191227153150
210. Qu J, Chen Y, Luo G, Zhong H, Xiao W, Yin H. Delirium in the Acute Phase of Ischemic Stroke: Incidence, Risk Factors, and Effects on Functional Outcome. *Journal of stroke and cerebrovascular diseases : the official journal of National Stroke Association*. Oct 2018;27(10):2641-2647. doi:10.1016/j.jstrokecerebrovasdis.2018.05.034
211. Racine AM, Fong TG, Travison TG, et al. Alzheimer's-related cortical atrophy is associated with postoperative delirium severity in persons without dementia. *Neurobiology of aging*. Nov 2017
2017-10-08 2017;59:55-63. doi:http://dx.doi.org/10.1016/j.neurobiolaging.2017.07.010
212. Radinovic K, Markovic-Denic L, Dubljanin-Raspopovic E, Marinkovic J, Milan Z, Bumbasirevic V. Estimating the effect of incident delirium on short-term outcomes in aged hip fracture patients through propensity score analysis. *Geriatrics & gerontology international*. Jul 2015
2017-09-26 2015;15(7):848-855. doi:http://dx.doi.org/10.1111/ggi.12358
213. Radinovic K, Denic LM, Milan Z, Cirkovic A, Baralic M, Bumbasirevic V. Impact of intraoperative blood pressure, blood pressure fluctuation, and pulse pressure on postoperative delirium in elderly patients with hip fracture: A prospective cohort study. *INJURY-INTERNATIONAL JOURNAL OF THE CARE OF THE INJURED*. 2019/09//undefined 2019;50(9):1558-1564. doi:10.1016/j.injury.2019.06.026

© 2023 Ormseth CH et al. *JAMA Network Open.*

214. Ranhoff AH, Rozzini R, Sabatini T, Cassinadri A, Boffelli S, Trabucchi M. Delirium in a sub-intensive care unit for the elderly: occurrence and risk factors. *Aging clinical and experimental research*. Oct 2006;18(5):440-5.
215. Rao A, Shi SM, Afilalo J, et al. Physical Performance and Risk of Postoperative Delirium in Older Adults Undergoing Aortic Valve Replacement. *CLINICAL INTERVENTIONS IN AGING*. 2020 2020;15:1471-1479. doi:10.2147/CIA.S257079
216. Ren Q, Wen Y-Z, Wang J, et al. Elevated Level of Serum C-reactive Protein Predicts Postoperative Delirium among Patients Receiving Cervical or Lumbar Surgery. *BioMed research international*. 2020 2020;2020:5480148. doi:10.1155/2020/5480148
217. Ritchie CW, Newman TH, Leurent B, Sampson EL. The association between C-reactive protein and delirium in 710 acute elderly hospital admissions. *International psychogeriatrics*. May 2014;26(5):717-24. doi:10.1017/s1041610213002433
218. Rizzi MA, Torres Bonafonte OH, Alquezar A, et al. Prognostic value and risk factors of delirium in emergency patients with decompensated heart failure. *Journal of the American Medical Directors Association*. Sep 1 2015;16(9):799.e1-6. doi:10.1016/j.jamda.2015.06.006
219. Robinson TN, Raeburn CD, Tran ZV, Angles EM, Brenner LA, Moss M. Postoperative delirium in the elderly: risk factors and outcomes. *Annals of surgery*. Jan 2009;249(1):173-8. doi:10.1097/SLA.0b013e31818e4776
220. Roggenbach J, Klamann M, von Haken R, Bruckner T, Karck M, Hofer S. Sleep-disordered breathing is a risk factor for delirium after cardiac surgery: a prospective cohort study. *Critical care (London, England)*. Sep 5 2014;18(5):477. doi:10.1186/s13054-014-0477-1
221. Rudolph JL, Jones RN, Grande LJ, et al. Impaired executive function is associated with delirium after coronary artery bypass graft surgery. *Journal of the American Geriatrics Society*. Jun 2006;54(6):937-41. doi:10.1111/j.1532-5415.2006.00735.x
222. Rudolph JL, Jones RN, Rasmussen LS, Silverstein JH, Inouye SK, Marcantonio ER. Independent vascular and cognitive risk factors for postoperative delirium. *The American journal of medicine*. Sep 2007;120(9):807-13. doi:10.1016/j.amjmed.2007.02.026
223. Rudolph JL, Babikian VL, Treanor P, et al. Microemboli are not associated with delirium after coronary artery bypass graft surgery. *Perfusion*. Nov 2009;24(6):409-15. doi:10.1177/0267659109358207
224. Saczynski JS, Inouye SK, Kosar CM, et al. Cognitive and brain reserve and the risk of postoperative delirium in older patients: analysis of data from a prospective observational study. *Lancet Psychiatry*. Nov 2014;1(6):437-43. doi:10.1016/s2215-0366(14)00009-1
225. Sanchez-Hurtado LA, Hernandez-Sanchez N, Del Moral-Armengol M, et al. Incidence of Delirium in Critically Ill Cancer Patients. *Pain research & management*. 2018;2018:4193275. doi:10.1155/2018/4193275
226. Santos FS, Velasco IT, Fraguas R, Jr. Risk factors for delirium in the elderly after coronary artery bypass graft surgery. *International psychogeriatrics*. Jun 2004;16(2):175-93.
227. Schoen J, Meyerrose J, Paarmann H, Heringlake M, Hueppe M, Berger KU. Preoperative regional cerebral oxygen saturation is a predictor of postoperative delirium in on-pump cardiac surgery patients: a prospective observational trial. *Critical care (London, England)*. 2011;15(5):R218. doi:10.1186/cc10454
228. Schor JD, Levkoff SE, Lipsitz LA, et al. Risk factors for delirium in hospitalized elderly. *Jama*. Feb 12 1992;267(6):827-31.

© 2023 Ormseth CH et al. *JAMA Network Open.*

229. Schreiber MP, Colantuoni E, Bienvenu OJ, et al. Corticosteroids and transition to delirium in patients with acute lung injury. *Critical care medicine*. Jun 2014;42(6):1480-6. doi:10.1097/ccm.0000000000000247
230. Serafim RB, Dutra MF, Saddy F, et al. Delirium in postoperative nonventilated intensive care patients: risk factors and outcomes. *Ann Intensive Care*. Dec 31 2012;2(1):51. doi:10.1186/2110-5820-2-51
231. Seymour CW, Pandharipande PP, Koestner T, et al. Diurnal sedative changes during intensive care: Impact on liberation from mechanical ventilation and delirium. *Critical care medicine*. Oct 2012;40(10):2788-2796. doi:10.1097/CCM.0b013e31825b8ade
232. Shen H, Shao Y, Chen J, Guo J. Insulin-Like Growth Factor-1, a Potential Predicative Biomarker for Postoperative Delirium Among Elderly Patients with Open Abdominal Surgery. *Current pharmaceutical design*. 2016;22(38):5879-5883. doi:10.2174/1381612822666160813234311
233. Sheng AZ, Shen Q, Cordato D, Zhang YY, Yin Chan DK. Delirium within three days of stroke in a cohort of elderly patients. *Journal of the American Geriatrics Society*. Aug 2006;54(8):1192-8. doi:10.1111/j.1532-5415.2006.00806.x
234. Shim J, DePalma G, Sands LP, Leung JM. Prognostic Significance of Postoperative Subsyndromal Delirium. *Psychosomatics*. Nov-Dec 2015;56(6):644-51. doi:10.1016/j.psym.2015.05.002
235. Shin JE, Kyeong S, Lee JS, et al. A personality trait contributes to the occurrence of postoperative delirium: a prospective study. *BMC psychiatry*. Nov 3 2016;16(1):371. doi:10.1186/s12888-016-1079-z
236. Shioiri A, Kurumaji A, Takeuchi T, Matsuda H, Arai H, Nishikawa T. White matter abnormalities as a risk factor for postoperative delirium revealed by diffusion tensor imaging. *The American journal of geriatric psychiatry : official journal of the American Association for Geriatric Psychiatry*. Aug 2010;18(8):743-53. doi:10.1097/JGP.0b013e3181d145c5
237. Shioiri A, Kurumaji A, Takeuchi T, Nemoto K, Arai H, Nishikawa T. A Decrease in the Volume of Gray Matter as a Risk Factor for Postoperative Delirium Revealed by an Atlas-based Method. *The American journal of geriatric psychiatry : official journal of the American Association for Geriatric Psychiatry*. Jul 2016;24(7):528-36. doi:10.1016/j.jagp.2015.09.002
238. Sieber FE, Mears S, Lee H, Gottschalk A. Postoperative Opioid Consumption and Its Relationship to Cognitive Function in Older Adults with Hip Fracture. *Journal of the American Geriatrics Society*. Dec 2011;59(12):2256-2262. doi:10.1111/j.1532-5415.2011.03729.x
239. Siew ED, Fissell WH, Tripp CM, et al. Acute Kidney Injury as a Risk Factor for Delirium and Coma during Critical Illness. *American journal of respiratory and critical care medicine*. Jun 15 2017;195(12):1597-1607. doi:10.1164/rccm.201603-0476OC
240. Singler K, Thiem U, Christ M, et al. Aspects and assessment of delirium in old age First data from a German interdisciplinary emergency department. *Zeitschrift fur Gerontologie und Geriatrie*. Dec 2014;47(8):680-685. doi:10.1007/s00391-014-0615-z
241. Slatore CG, Goy ER, O'Hearn D J, et al. Sleep quality and its association with delirium among veterans enrolled in hospice. *The American journal of geriatric psychiatry : official journal of the American Association for Geriatric Psychiatry*. Apr 2012;20(4):317-26. doi:10.1097/JGP.0b013e3182487680

© 2023 Ormseth CH et al. *JAMA Network Open.*

242. Slor CJ, de Jonghe JF, Vreeswijk R, et al. Anesthesia and postoperative delirium in older adults undergoing hip surgery. *Journal of the American Geriatrics Society*. Jul 2011;59(7):1313-9. doi:10.1111/j.1532-5415.2011.03452.x
243. Slor CJ, Witlox J, Adamis D, et al. The trajectory of C-reactive protein serum levels in older hip fracture patients with postoperative delirium. *INTERNATIONAL JOURNAL OF GERIATRIC PSYCHIATRY*. 2019/10//undefined 2019;34(10):1438-1446. doi:10.1002/gps.5139
244. Smulter N, Lingehall HC, Gustafson Y, Olofsson B, Engstrom KG. Delirium after cardiac surgery: incidence and risk factors. *Interactive cardiovascular and thoracic surgery*. Nov 2013;17(5):790-6. doi:10.1093/icvts/ivt323
245. Smulter N, Lingehall HC, Gustafson Y, et al. Disturbances in Oxygen Balance During Cardiopulmonary Bypass: A Risk Factor for Postoperative Delirium. *Journal of cardiothoracic and vascular anesthesia*. Apr 2018;32(2):684-690. doi:10.1053/j.jvca.2017.08.035
246. Soehle M, Dittmann A, Ellerkmann RK, Baumgarten G, Putensen C, Guenther U. Intraoperative burst suppression is associated with postoperative delirium following cardiac surgery: a prospective, observational study. *BMC anesthesiology*. Apr 2015;1561. doi:10.1186/s12871-015-0051-7
247. Soh S, Shim J-K, Song J-W, Choi N, Kwak Y-L. Preoperative transcranial Doppler and cerebral oximetry as predictors of delirium following valvular heart surgery: a case-control study. *Journal of clinical monitoring and computing*. 2020/08//undefined 2020;34(4):715-723. doi:10.1007/s10877-019-00385-x
248. Sosa FA, Roberti J, Franco MT, Kleinert MM, Patron AR, Osatnik J. Assessment of delirium using the PRE-DELIRIC model in an intensive care unit in Argentina. *Revista Brasileira de terapia intensiva*. Mar 2018;30(1):50-56. Avaliacao de delirium com uso do modelo PRE-DELIRIC em uma unidade de terapia intensiva na Argentina. doi:10.5935/0103-507x.20180010
249. Srinonprasert V, Pakdeewongse S, Assanasen J, et al. Risk factors for developing delirium in older patients admitted to general medical wards. *Journal of the Medical Association of Thailand = Chotmaihet thangphaet*. Feb 2011;94 Suppl 1:S99-104.
250. Štubljar D, Štefin M, Tacar MP, Cerović O, Grosek Š. Prolonged hospitalization is a risk factor for delirium onset: one-day prevalence study in Slovenian INTENSIVE CARE UNITS. *Acta clinica Croatica*. 2019/06//undefined 2019;58(2):265-273. doi:10.20471/acc.2019.58.02.09
251. Styra R, Larsen E, Dimas MA, et al. The effect of preoperative cognitive impairment and type of vascular surgery procedure on postoperative delirium with associated cost implications. *Journal of vascular surgery*. Jan 2019;69(1):201-209. doi:10.1016/j.jvs.2018.05.001
252. Susano MJ, Grasfield RH, Friese M, et al. Brief Preoperative Screening for Frailty and Cognitive Impairment Predicts Delirium after Spine Surgery. *Anesthesiology*. 2020/12/01/ 2020;133(6):1184-1191. doi:10.1097/ALN.0000000000003523
253. Tahir M, Malik SS, Ahmed U, Kozdryk J, Naqvi SH, Malik A. Risk factors for onset of delirium after neck of femur fracture surgery: a prospective observational study. *Sicot-J*. Jul 2018;4Unsp 27. doi:10.1051/sicotj/2018018
254. Taipale PG, Ratner PA, Galdas PM, et al. The association between nurse-administered midazolam following cardiac surgery and incident delirium: an observational study. *International journal of nursing studies*. Sep 2012;49(9):1064-73. doi:10.1016/j.ijnurstu.2012.03.008

© 2023 Ormseth CH et al. *JAMA Network Open.*

255. Tan MC, Felde A, Kuskowski M, et al. Incidence and predictors of post-cardiotomy delirium. *The American journal of geriatric psychiatry : official journal of the American Association for Geriatric Psychiatry*. Jul 2008;16(7):575-83. doi:10.1097/JGP.0b013e318172b418
256. Tao L, Xiaodong X, Qiang M, Jiao L, Xu Z. Prediction of postoperative delirium by comprehensive geriatric assessment among elderly patients with hip fracture. Article in Press. *Irish Journal of Medical Science*. 2019;doi:10.1007/s11845-019-02011-w
257. Thillainadesan J, Mudge AM, Aitken SJ, et al. The Prognostic Performance of Frailty for Delirium and Functional Decline in Vascular Surgery Patients. *Journal of the American Geriatrics Society*. 2021/03//undefined 2021;69(3):688-695. doi:10.1111/jgs.16907
258. Thisayakorn P, Tangwongchai S, Tantavisut S, et al. Immune, Blood Cell, and Blood Gas Biomarkers of Delirium in Elderly Individuals with Hip Fracture Surgery. *DEMENTIA AND GERIATRIC COGNITIVE DISORDERS*. 2021/08//undefined 2021;50(2):161-169. doi:10.1159/000517510
259. Theologou S, Giakoumidakis K, Charitos C. Perioperative predictors of delirium and incidence factors in adult patients post cardiac surgery. *Pragmatic and Observational Research*. 2018;9:11-19. doi:10.2147/por.S157909
260. Tiwary N, Treggiari MM, Yanez ND, et al. Agreement Between the Mini-Cog in the Preoperative Clinic and on the Day of Surgery and Association With Postanesthesia Care Unit Delirium: A Cohort Study of Cognitive Screening in Older Adults. *Anesthesia and analgesia*. 2021/04/01/ 2021;132(4):1112-1119. doi:10.1213/ANE.0000000000005197
261. Todd OM, Gelrich L, MacLullich AM, Driessen M, Thomas C, Kreisel SH. Sleep Disruption at Home As an Independent Risk Factor for Postoperative Delirium. *Journal of the American Geriatrics Society*. May 2017;65(5):949-957. doi:10.1111/jgs.14685
262. Tong CY, Huang CY, Wu JX, Xu MY, Cao H. The Prevalence and Impact of Undiagnosed Mild Cognitive Impairment in Elderly Patients Undergoing Thoracic Surgery: A Prospective Cohort Study. *JOURNAL OF CARDIOTHORACIC AND VASCULAR ANESTHESIA*. 2020/09//undefined 2020;34(9):2413-2418. doi:10.1053/j.jvca.2020.03.011
263. Tow A, Holtzer R, Wang C, et al. Cognitive Reserve and Postoperative Delirium in Older Adults. *Journal of the American Geriatrics Society*. Jun 2016;64(6):1341-6. doi:10.1111/jgs.14130
264. Tripp BA, Dillon ST, Yuan M, et al. Targeted metabolomics analysis of postoperative delirium. *SCIENTIFIC REPORTS*. 2021/01/15/ 2021;11(1)doi:10.1038/s41598-020-80412-z
265. Tsuruta R, Oda Y, Shintani A, et al. Delirium and coma evaluated in mechanically ventilated patients in the intensive care unit in Japan: a multi-institutional prospective observational study. *Journal of critical care*. Jun 2014;29(3):472.e1-5. doi:10.1016/j.jcrc.2014.01.021
266. Tully PJ, Baker RA, Winefield HR, Turnbull DA. Depression, anxiety disorders and Type D personality as risk factors for delirium after cardiac surgery. *The Australian and New Zealand journal of psychiatry*. Nov 2010;44(11):1005-11. doi:10.3109/00048674.2010.495053
267. Uchida M, Okuyama T, Ito Y, et al. Prevalence, course and factors associated with delirium in elderly patients with advanced cancer: a longitudinal observational study. *Japanese journal of clinical oncology*. Oct 2015;45(10):934-40. doi:10.1093/jjco/hyv100

© 2023 Ormseth CH et al. *JAMA Network Open.*

268. Uguz F, Kayrak M, Cicek E, Kayhan F, Ari H, Altunbas G. Delirium following acute myocardial infarction: incidence, clinical profiles, and predictors. *Perspectives in psychiatric care*. Apr 2010;46(2):135-42. doi:10.1111/j.1744-6163.2010.00249.x
269. van der Mast RC, van den Broek WW, Fekkes D, Pepplinkhuizen L, Habbema JD. Is delirium after cardiac surgery related to plasma amino acids and physical condition? *The Journal of neuropsychiatry and clinical neurosciences*. Winter 2000;12(1):57-63. doi:10.1176/jnp.12.1.57
270. van der Wulp K, van Wely M, van Heijningen L, et al. Delirium After Transcatheter Aortic Valve Implantation Under General Anesthesia: Incidence, Predictors, and Relation to Long-Term Survival. *JOURNAL OF THE AMERICAN GERIATRICS SOCIETY*. 2019/11//undefined 2019;67(11):2325-2330. doi:10.1111/jgs.16087
271. Van Grootven B, Detroyer E, Devriendt E, et al. Is preoperative state anxiety a risk factor for postoperative delirium among elderly hip fracture patients? *Geriatrics & gerontology international*. Aug 2016;16(8):948-955. doi:10.1111/ggi.12581
272. van Munster BC, Korevaar JC, Zwinderman AH, Levi M, Wiersinga WJ, De Rooij SE. Time-course of cytokines during delirium in elderly patients with hip fractures. *Journal of the American Geriatrics Society*. Sep 2008;56(9):1704-9. doi:10.1111/j.1532-5415.2008.01851.x
273. van Munster BC, Korevaar JC, Korse CM, Bonfrer JM, Zwinderman AH, de Rooij SE. Serum S100B in elderly patients with and without delirium. *International journal of geriatric psychiatry*. Mar 2010;25(3):234-9. doi:10.1002/gps.2326
274. van Munster BC, Yazdanpanah M, Tanck MW, et al. Genetic polymorphisms in the DRD2, DRD3, and SLC6A3 gene in elderly patients with delirium. *American journal of medical genetics Part B, Neuropsychiatric genetics : the official publication of the International Society of Psychiatric Genetics*. Jan 5 2010;153b(1):38-45. doi:10.1002/ajmg.b.30943
275. van Munster BC, Bisschop PH, Zwinderman AH, et al. Cortisol, interleukins and S100B in delirium in the elderly. *Brain and cognition*. Oct 2010;74(1):18-23. doi:10.1016/j.bandc.2010.05.010
276. van Munster BC, Thomas C, Kreisel SH, et al. Longitudinal assessment of serum anticholinergic activity in delirium of the elderly. *Journal of psychiatric research*. Oct 2012;46(10):1339-45. doi:10.1016/j.jpsychires.2012.06.015
277. Vasunilashorn SM, Dillon ST, Inouye SK, et al. High C-Reactive Protein Predicts Delirium Incidence, Duration, and Feature Severity After Major Noncardiac Surgery. *Journal of the American Geriatrics Society*. Aug 2017;65(8):e109-e116. doi:10.1111/jgs.14913
278. Vasunilashorn SM, Ngo LH, Jones RN, et al. The Association Between C-Reactive Protein and Postoperative Delirium Differs by Catechol-O-Methyltransferase Genotype. *American Journal of Geriatric Psychiatry*. Jan 2019;27(1):1-8. doi:10.1016/j.jagp.2018.09.007
279. Vaurio LE, Sands LP, Wang Y, Mullen EA, Leung JM. Postoperative delirium: the importance of pain and pain management. *Anesthesia and analgesia*. Apr 2006;102(4):1267-73. doi:10.1213/01.ane.0000199156.59226.af
280. Veliz-Reissmuller G, Aguero Torres H, van der Linden J, Lindblom D, Eriksdotter Jonhagen M. Pre-operative mild cognitive dysfunction predicts risk for post-operative delirium after elective cardiac surgery. *Aging clinical and experimental research*. Jun 2007;19(3):172-7.

© 2023 Ormseth CH et al. *JAMA Network Open.*

281. Verloo H, Goulet C, Morin D, von Gunten A. Association between frailty and delirium in older adult patients discharged from hospital. *Clinical interventions in aging*. 2016;11:55-63. doi:10.2147/cia.S100576
282. Villalpando-Berumen JM, Pineda-Colorado AM, Palacios P, Reyes-Guerrero J, Villa AR, Gutierrez-Robledo LM. Incidence of delirium, risk factors, and long-term survival of elderly patients hospitalized in a medical specialty teaching hospital in Mexico City. *International psychogeriatrics*. Dec 2003;15(4):325-36.
283. Vondeling AM, Knol W, Egberts TCG, Slooter AJC. Anticholinergic drug exposure at intensive care unit admission affects the occurrence of delirium. A prospective cohort study. *EUROPEAN JOURNAL OF INTERNAL MEDICINE*. 2020/08//undefined 2020;78:121-126. doi:10.1016/j.ejim.2020.04.062
284. Wada S, Inoguchi H, Sadahiro R, et al. Preoperative Anxiety as a Predictor of Delirium in Cancer Patients: A Prospective Observational Cohort Study. *World journal of surgery*. Jan 2019;43(1):134-142. doi:10.1007/s00268-018-4761-0
285. Wan TT, Wei PH, Yao Y, Liu H, Li JJ. Association of Carotid Plaque and Serum Lipoprotein-Associated Phospholipase A2 (LP-PLA2) with Postoperative Delirium in Geriatric Patients Undergoing Hip Replacement: A Prospective Cohort Study. *MEDICAL SCIENCE MONITOR*. 2020/11/14/ 2020;26doi:10.12659/MSM.927763
286. Wang J, Ji Y, Wang N, et al. Risk factors for the incidence of delirium in cerebrovascular patients in a Neurosurgery Intensive Care Unit: A prospective study. *Journal of clinical nursing*. Jan 2018;27(1-2):407-415. doi:10.1111/jocn.13943
287. Wang CM, Huang HW, Wang YM, et al. Incidence and risk factors of postoperative delirium in patients admitted to the ICU after elective intracranial surgery A prospective cohort study. *EUROPEAN JOURNAL OF ANAESTHESIOLOGY*. 2020/01//undefined 2020;37(1):14-24. doi:10.1097/EJA.0000000000001074
288. Wang J, Ji YY, Wang N, et al. Establishment and validation of a delirium prediction model for neurosurgery patients in intensive care. *INTERNATIONAL JOURNAL OF NURSING PRACTICE*. 2020/08//undefined 2020;26(4)doi:10.1111/ijn.12818
289. Wang G, Zhang L, Qi Y, et al. Development and Validation of a Postoperative Delirium Prediction Model for Elderly Orthopedic Patients in the Intensive Care Unit. *JOURNAL OF HEALTHCARE ENGINEERING*. 2021/06/08/ 2021;2021doi:10.1155/2021/9959077
290. Wang M-L, Min J, Sands LP, Leung JM. Midazolam Premedication Immediately Before Surgery Is Not Associated With Early Postoperative Delirium. *Anesthesia and analgesia*. 2021/09/01/ 2021;133(3):765-771. doi:10.1213/ANE.0000000000005482
291. Watne LO, Hall RJ, Molden E, et al. Anticholinergic activity in cerebrospinal fluid and serum in individuals with hip fracture with and without delirium. *Journal of the American Geriatrics Society*. Jan 2014;62(1):94-102. doi:10.1111/jgs.12612
292. Watne LO, Idland AV, Fekkes D, et al. Increased CSF levels of aromatic amino acids in hip fracture patients with delirium suggests higher monoaminergic activity. *BMC geriatrics*. Aug 2 2016;16:149. doi:10.1186/s12877-016-0324-0
293. Wesselink EM, Kappen TH, Van Klei WA, Dieleman JM, Van Dijk D, Slooter AJC. Intraoperative hypotension and delirium after on-pump cardiac surgery. Article. *British journal of anaesthesia*. 2015;115(3):427-433. doi:10.1093/bja/aev256

© 2023 Ormseth CH et al. *JAMA Network Open.*

294. Wilson K, Broadhurst C, Diver M, Jackson M, Mottram P. Plasma insulin growth factor-1 and incident delirium in older people. *International journal of geriatric psychiatry*. Feb 2005;20(2):154-9. doi:10.1002/gps.1265
295. Witlox J, Kalisvaart KJ, de Jonghe JF, et al. Cerebrospinal fluid beta-amyloid and tau are not associated with risk of delirium: a prospective cohort study in older adults with hip fracture. *Journal of the American Geriatrics Society*. Jul 2011;59(7):1260-7. doi:10.1111/j.1532-5415.2011.03482.x
296. Witlox J, Adamis D, Koenderman L, et al. Preoperative Cerebrospinal Fluid Cortisol and the Risk of Postoperative Delirium: A Prospective Study of Older Hip Fracture Patients. *Dementia and geriatric cognitive disorders*. 2020 2020;49(6):604-610. doi:10.1159/000512984
297. Wood MD, Maslove DM, Muscedere JG, Day AG, Gordon Boyd J. Low brain tissue oxygenation contributes to the development of delirium in critically ill patients: A prospective observational study. *Journal of critical care*. Oct 2017;41:289-295. doi:10.1016/j.jcrc.2017.06.009
298. Wu J, Gao S, Zhang S, et al. Perioperative risk factors for recovery room delirium after elective non-cardiovascular surgery under general anaesthesia.
299. Xie Z, Swain CA, Ward SA, et al. Preoperative cerebrospinal fluid beta-Amyloid/Tau ratio and postoperative delirium. *Annals of clinical and translational neurology*. May 1 2014;1(5):319-328. doi:10.1002/acn3.58
300. Xing J, Yuan Z, Jie Y, Liu Y, Wang M, Sun Y. Risk factors for delirium: Are therapeutic interventions part of it? Article. *Neuropsychiatric Disease and Treatment*. 2019;15:1321-1327. doi:10.2147/NDT.S192836
301. Xu W, Ma H, Li W, Zhang C. The risk factors of postoperative delirium in patients with hip fracture: implication for clinical management. *BMC musculoskeletal disorders*. 2021/03/07/ 2021;22(1):254. doi:10.1186/s12891-021-04091-1
302. Yam KK, Shea YF, Chan TC, et al. Prevalence and risk factors of delirium and subsyndromal delirium in Chinese older adults. *Geriatrics & gerontology international*. Dec 2018;18(12):1625-1628. doi:10.1111/ggi.13545
303. Yang FM, Inouye SK, Fearing MA, Kiely DK, Marcantonio ER, Jones RN. Participation in activity and risk for incident delirium. *Journal of the American Geriatrics Society*. Aug 2008;56(8):1479-84. doi:10.1111/j.1532-5415.2008.01792.x
304. Yen TE, Allen JC, Rivelli SK, et al. Association between Serum IGF-I levels and Postoperative Delirium in Elderly Subjects Undergoing Elective Knee Arthroplasty. *Scientific reports*. Feb 5 2016;6:20736. doi:10.1038/srep20736
305. Yoshimura Y, Kubo S, Shirata K, et al. Risk factors for postoperative delirium after liver resection for hepatocellular carcinoma. *World journal of surgery*. Oct 2004;28(10):982-6. doi:10.1007/s00268-004-7344-1
306. Yuan Y, Li ZQ, Yang N, et al. Exosome alpha-Synuclein Release in Plasma May be Associated With Postoperative Delirium in Hip Fracture Patients. *FRONTIERS IN AGING NEUROSCIENCE*. 2020/03/13/ 2020;12doi:10.3389/fnagi.2020.00067
307. Zaal IJ, Spruyt CF, Peelen LM, et al. Intensive care unit environment may affect the course of delirium. *Intensive care medicine*. Mar 2013;39(3):481-488. doi:10.1007/s00134-012-2726-6

© 2023 Ormseth CH et al. *JAMA Network Open.*

308. Zhang DF, Su X, Meng ZT, et al. Preoperative severe hypoalbuminemia is associated with an increased risk of postoperative delirium in elderly patients: Results of a secondary analysis. *Journal of critical care*. Apr 2018;44:45-50. doi:10.1016/j.jcrc.2017.09.182
309. Zhang Y, He S-T, Nie B, Li X-Y, Wang D-X. Emergence delirium is associated with increased postoperative delirium in elderly: a prospective observational study. *Journal of anesthesia*. 2020/10//undefined 2020;34(5):675-687. doi:10.1007/s00540-020-02805-8
310. Zhang F, He ST, Zhang Y, Mu DL, Wang DX. Malnutrition is not related with emergence delirium in older patients after noncardiac surgery. *BMC GERIATRICS*. 2021/05/17/ 2021;21(1)doi:10.1186/s12877-021-02270-2
311. Zhang R, Bai LF, Han XL, Huang SC, Zhou LT, Duan J. Incidence, characteristics, and outcomes of delirium in patients with noninvasive ventilation: a prospective observational study. *BMC PULMONARY MEDICINE*. 2021/05/11/ 2021;21(1)doi:10.1186/s12890-021-01517-3
312. Zhao Y, Xia X, Xie D, et al. Geriatric Nutritional Risk Index can predict postoperative delirium and hospital length of stay in elderly patients undergoing non-cardiac surgery. *Geriatrics & gerontology international*. 2020/08//undefined 2020;20(8):759-764. doi:10.1111/ggi.13963
313. Zhao YL, Yue JR, Lei P, et al. Neutrophil-lymphocyte ratio as a predictor of delirium in older internal medicine patients: a prospective cohort study. *BMC GERIATRICS*. 2021/05/25/ 2021;21(1)doi:10.1186/s12877-021-02284-w
314. Zipprich HM, Arends MC, Schumacher U, et al. Outcome of Older Patients with Acute Neuropsychological Symptoms Not Fulfilling Criteria of Delirium. *JOURNAL OF THE AMERICAN GERIATRICS SOCIETY*. 2020/07//undefined 2020;68(7):1469-1475. doi:10.1111/jgs.16422
315. Zrour C, Haddad R, Zoghbi M, Kharsa Z, Hijazi M, Naja W. Prospective, multi-centric benchmark study assessing delirium: prevalence, incidence and its correlates in hospitalized elderly Lebanese patients. *AGING CLINICAL AND EXPERIMENTAL RESEARCH*. 2020/04//undefined 2020;32(4):689-697. doi:10.1007/s40520-019-01242-2

© 2023 Ormseth CH et al. *JAMA Network Open.*

eFigure. Study Flowchart

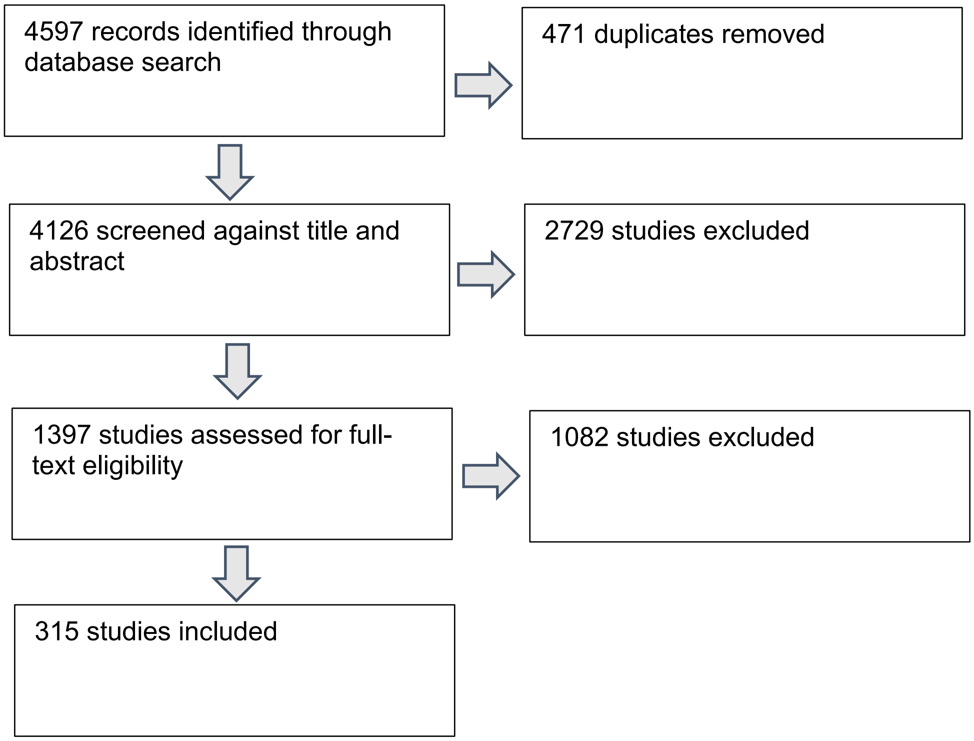

© 2023 Ormseth CH et al. *JAMA Network Open.*
