# Supplementary material for: Predisposing and Precipitating Factors Associated With Delirium: A Systematic Review
Source: JAMA Netw Open. 2023 Jan 6;6(1):e2249950. doi: 10.1001/jamanetworkopen.2022.49950 (PMC9856673; doi:10.1001/jamanetworkopen.2022.49950)
Supplement: Supplement 2. — Data Sharing Statement [file jamanetwopen-e2249950-s002.pdf]

Data Sharing Statement

Ormseth CH, LaHue SC, Oldham MA, Josephson SA, Whitaker E, Douglas VC. Predisposing and precipitating factors associated with delirium: a systematic review. *JAMA Netw Open*. 2023;6(1):e2249950 doi:10.1001/jamanetworkopen.2022.49950

Data
Data available: No

© 2023 Ormseth CH et al. *JAMA Network Open*.
